# *Ralstonia solanacearum* type III effector RipAF1 mediates plant resistance signaling by ADP-ribosylation of host FBN1

**DOI:** 10.1093/hr/uhae162

**Published:** 2024-06-12

**Authors:** Wei Wu, Huasong Zou, Huiying Zheng, Xinyu Chen, Xuming Luo, Xiaojing Fan, Tao Zhuo, Weiguo Miao

**Affiliations:** Sanya Institute of Breeding and Multiplication/School of Tropical Agriculture and Forestry, Hainan University, Haikou 570228, China; College of Plant Protection, Fujian Agriculture and Forestry University, Fuzhou, 350002, China; School of Life and Health Sciences, Huzhou College, Huzhou, Zhejiang, 313000, China; Sanya Institute of Breeding and Multiplication/School of Tropical Agriculture and Forestry, Hainan University, Haikou 570228, China; College of Plant Protection, Fujian Agriculture and Forestry University, Fuzhou, 350002, China; State Key Laboratory of Plant Genomics, Institute of Microbiology, Chinese Academy of Sciences, Beijing, 100101, China; College of Plant Protection, Fujian Agriculture and Forestry University, Fuzhou, 350002, China; College of Plant Protection, Fujian Agriculture and Forestry University, Fuzhou, 350002, China; Sanya Institute of Breeding and Multiplication/School of Tropical Agriculture and Forestry, Hainan University, Haikou 570228, China

## Abstract

*Ralstonia solanacearum* (*Rso*) causes destructive bacterial wilt across a broad range of host plants by delivering a repertoire of type III effectors. In the present study, we determined that the deletion of the type III effector RipAF1 resulted in increased virulence on *Nicotiana benthamiana*, *Solanum lycopersicum*, and *Capsicum annuum* plants. RipAF1 showed ADP-ribosylation activity *in vivo* and *in vitro*. Transient overexpression of RipAF1 suppressed jasmonic acid (JA) signaling and induced salicylic acid (SA) signaling. The ADP-ribosylation activity of RipAF1 was essential for JA and SA signaling mediation. Host fibrillin FBN1 was identified as a RipAF1-interactor that is ADP-ribosylated by RipAF1 directly. Most importantly, the ADP-ribosylation of conserved residues of FBN1 contributes to its localization to the plasma membrane and leads to the suppression of JA signaling and induction of SA signaling. We concluded that RipAF1 mediates antagonistic crosstalk between JA and SA signaling pathways by ADP-ribosylation of FBN1.

## Introduction

The beta proteobacterium *Ralstonia solanacearum* (*Rso*) infects a wide range of host plant species, causing devastating bacterial wilt disease worldwide [1]. *Rso* attaches to and penetrates into host roots by means of numerous infection mechanisms, including secretion of cell wall-degrading enzymes, biofilm formation, and production of exopolysaccharides [2]. Furthermore, *Rso* injects a set of type III effectors (T3Es) into host cells to modulate defense or susceptible reactions via a type III secretion system [3]. Through whole-genome data analysis, 102 *Ralstonia* injected proteins (Rips) and 16 hypothetical T3E genes have been successfully identified from among 140 *Rso* species [4]. As Rips have diverse roles in virulence, the pan-effectome of *Rso* strains results in a particular host width and specificity during infection [5].

The plant hormone jasmonic acid (JA) is a universal resistance signaling molecule that responds to necrotrophic pathogens in plants, whereas salicylic acid (SA) induces resistance to biotrophic and hemibiotrophic pathogens [6]. *Rso* is a hemibiotrophic bacterial pathogen with initial biotrophy followed by necrotrophy. Inoculation of susceptible pepper plants with *Rso* RS1002 leads to increased biosynthesis of JA and jasmonoyl-isoleucine (JA-Ile) and reduced biosynthesis of SA [7]. Knock-down of the pathogenesis-related gene *PR1* decreases tomato resistance to bacterial wilt disease [8]. Exogenous application of methyl jasmonate (MeJA) enhances short-term tolerance and then promotes disease susceptibility in the legume *Medicago truncatula* [9], while pretreating tomato plants with SA is beneficial for resistance against *Rso* [10]*.* JA and SA signaling are disturbed by a number of *Rso* Rips, including RipI, RipE1, and RipAB [11–13]. Among them, RipAB disrupts SA signaling by inhibiting *Arabidopsis* TGA transcription factors to achieve successful infection [13].

The ADP-ribosylation represents a post-translational modification affecting protein conformation, stability, activity, and subcellular localization [14]. The type III effector RipAF1 in *Rso* is a putative ADP-ribosyltransferase (ADP-RT) that was identified as a Rip from the *Rso* RS1000 strain [15]. RipAF1 is localized to the cytoplasm, cell membrane, and nucleus of host cells [16]. Its two homologous AvrPphFs (HopF1 and HopF2) in *Pseudomonas syringae* pv. *phaseolicola* are required for avirulence of the bacteria in bean cultivars carrying the *R1* gene [17]. Introduction of HopF2 into the *hopF2* mutant of *P. syringae* pv. *tomato* DC3000 enhanced bacterial growth in *Arabidopsis*. Expression of HopF2 in *P. syringae* pv. *tabaci* induced a HR reaction in W38 tobacoo plants [18]. AvrPphF-mediated defense signaling has been well documented in association with the HopF2 homolog in *P. syringae* pv. *tomato* DC3000 [19]. HopF2 is able to ADP-ribosylate *Arabidopsis thaliana* MAP kinase kinase 5 (MKK5), thus inhibiting the kinase activity that is essential for the activation of the MEKK1/MEKKs-MKK4/5-MPK3/6 cascade [18]. The arginine and aspartic acid residues at amino acid positions 71 and 175 of HopF2 are necessary for the interaction with MKK5 to block MAP kinase activation, resulting in suppression of innate immunity [18]. Two other independent studies have reported that HopF2 targets BAK1 and RIN4 proteins to suppress plant innate immunity and promote bacterial virulence [20, 21]. However, RipAF1 from *Rso* shows no distinct effect on suppressing the flg22-triggered innate defense reaction, suggesting it has a different role from that of HopF2 [22].

Fibrillin (FBN) family proteins are widely found in photosynthetic organisms ranging from cyanobacteria to vascular plants. Based on a phylogenetic analysis of sequence conservation and diversity, the FBNs from 10 photosynthetic eukaryotes can be classified into 11 clades, which include the taxa *Synechocystis*, *Chlamydomonas reinhardtii*, *Oryza sativa*, and *A. thaliana*. The expression of FBNs has been characterized in responses to different stress stimuli, as well as a close relationship with JA or ABA signaling [23–28]. The FBNs in various plastids are involved in the biogenesis of chloroplast plastoglobules, and those in thylakoids and stroma are involved in the storage, transport, and synthesis of lipid molecules for photoprotective functions against high-light stress [24, 29, 30]. Several FBNs play special roles in biotic stress responses. Knock-down of the fibrillin gene *FBN1* in *Lycopersicon esculentum* resulted in increased susceptibility to the necrotrophic fungus *Botrytis cinerea* [31]. The *FBN1b* deletion mutant of *Arabidopsis* is more sensitive to the virulent bacterium *P. syringae* [32]. *A. thaliana fib4* mutants and apple *fib4* RNA-interference transformants exhibited increased susceptibility to the bacterial pathogens *P. syringae* pv. *tomato* and *Erwinia amylovora*, respectively [33]. However, the role of plant FBNs in the response to *Rso* infection remains unclear.

The purpose of this work was to explore the role of T3E RipAF1 from tobacco-pathogenic *Rso* FJ1003 [34]. In addition to describing its negative role in virulence, we report that RipAF1 regulates SA and JA signaling by ADP-ribosylation of host FBN1. The results not only provide a deeper and fuller understanding of the RipAF1-mediated signaling pathway, but also highlight the role of ADP-ribosylation in *Rso-*host interactions.

## Results

### RipAF1 interferes with the expression of host JA and SA signaling marker genes

Based on the total repertoire analysis of T3E sequences (http://www.ralsto-t3e.org/), RipAF1 was determined to be present in representative strains from *Ralstonia* phylotypes I, IIA, and III, but absent in three phylotype IV strains: PSI07, BDBR229, and *Ralstonia syzygii*. Among the four representative phylotype IIB strains, RipAF1 was found in Po82 but absent from IPO1609, Molk2, and UW551 (Fig. S1A, see online supplementary material). The gene encoding RipAF1 was located in the 1 996 333-bp megaplasmid of the FJ1003 strain. The full-length gene encodes a 350-amino-acid protein with high similarity to its homolog in GMI1000 encoded by *Rsp0822*, which is also located on a megaplasmid. Based on phylogenetic analysis of the inferred amino acid sequences, the evolution of RipAF1 is closely related to the phylotype in that RipAF1 sequences of the same phylotype were confirmed to be monophyletic (Fig. S1B, see online supplementary material). The RipAF1 sequence from FJ1003 was most closely related to GMI1000 RipAF1, and the two proteins were grouped together with the RipAF1 sequences from phylotype I strains (Fig. S1B, see online supplementary material). The RipAF1 has no transmembrane domain but possesses a putative ADP-ribosyltransferase (ADP-RT) domain from positions 155 to 338 (Fig. S1C, see online supplementary material). This domain shares 99% identity with the RipAF1 sequence from GMI1000 and 37% similarity with AvrPphF family HopF1 and HopF2 sequences from *Pseudomonas* (Fig.S1D, see online supplementary material), indicating that RipAF1 may show avirulence property on hosts.

**Figure 1:**
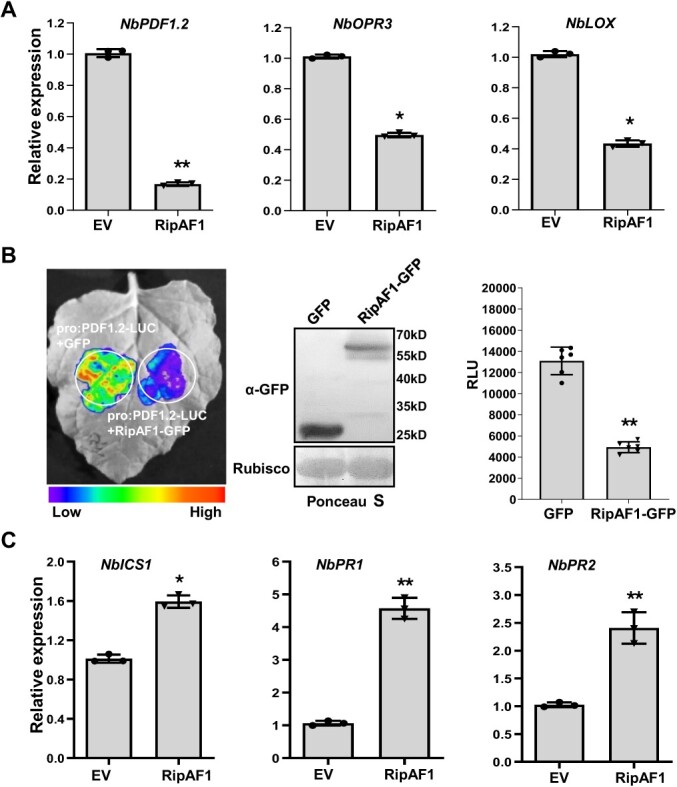
Effect of RipAF1 expression in *Nicotiana benthamiana* on jasmonic acid (JA) and salicylic acid (SA) signaling pathways. **A** The reduced expression levels of the JA marker genes *NbPDF1.2*, *NbOPR3,* and *NbLOX* in *N*. *benthamiana* plant expressing RipAF1. Total RNA was isolated from leaves 48 h after agroinfiltration. Expression levels were determined by qRT-PCR analysis and normalized to that of the empty vector pJL12 (EV). Values are means ± SD (*n* = 3 biological replicates; Student’s *t*-test, ^*^P < 0.05, ^**^P < 0.01). **B** Luciferase assays for the inhibition of *NbPDF1.2* promoter activity by RipAF1. *NbPDF1.2* promoter luciferase fusion (pro:PDF1.2-Luc) and RipAF1-GFP constructs were co-expressed in *N. benthamiana* leaves. Luciferase activity was measured with a CCD imaging system. The co-expression of pro:PDF1.2-Luc and GFP served as the negative control. The gels on the right show the expression of GFP and RipAF1-GFP in western blots using anti-GFP antibody. Quantitative assay of the luciferase signal of pro:PDF1.2-Luc. The signal was quantified with a microplate luminescence reader. Values are means ± SD (*n* = 6 biological replicates; Student’s *t*-test, ^**^P < 0.01). **C** The increased transcript levels of the SA signaling marker genes *NbICS1, NbPR1*, and *NbPR2* in *N*. *benthamiana* expressing RipAF1, performed as in (**A**). Values are means ± SD (*n* = 3 biological replicates; Student’s *t*-test, ^*^P < 0.05, ^**^P < 0.01). All experiments were replicated three times with similar results, and representative results are shown.

To determine whether RipAF1 affects the hormone signaling pathway, the transcript levels of the JA marker genes *NbPDF1.2*, *NbOPR3,* and *NbLOX* were quantified. The transcript levels of the three JA marker genes were all significantly decreased upon transient expression of RipAF1 (Fig. 1A). *PDF1.2* was the gene with the most reduced expression, with an over 80% reduction relative to the control. For further confirmation of this suppression, the *PDF1.2* promoter was fused with a luciferase reporter and co-expressed with RipAF1-GFP. Compared with the GFP control, the expression of RipAF1-GFP remarkably inhibited luciferase activity, by over 62% (Fig. 1B). As SA signaling acts antagonistically to JA during bacterial wilt development, the transcript levels of SA signaling marker genes, such as *NbICS1*, *NbPR1*, and *NbPR2*, were studied. As expected, their expression levels were induced by RipAF1 (Fig. 1C).

### Deletion of *ripAF1* results in enhanced virulence on hosts

**Figure 2:**
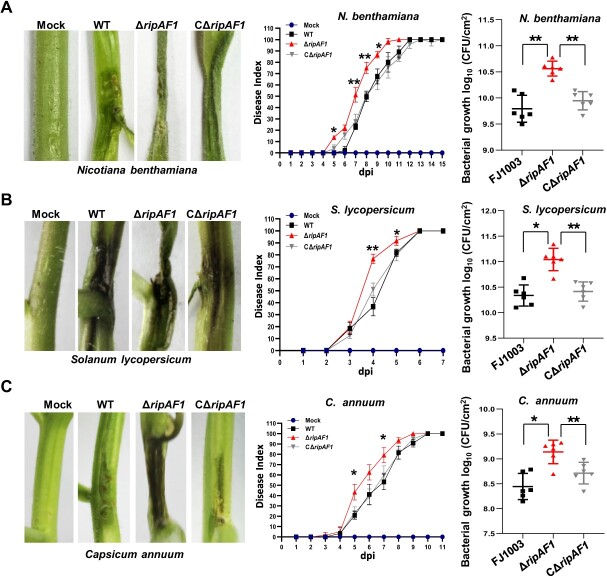
The enhanced virulence of the Δ*ripAF1* mutant of *Ralstonia solanacearum* FJ1003 on host plants. **A***Nicotiana benthamiana* plants inoculated with wild-type FJ1003, mutant Δ*ripAF1*, and the complemented strain CΔ*ripAF1.* Disease symptoms on stem were captured 7 d post-inoculation (dpi). The experiments were repeated three times with similar results, and representative results are shown. The disease index was assessed after stem inoculation. Each time point represents the mean disease severity of 24 inoculated plants per treatment. Error bars represent the standard deviation of three independent experiments (two-way ANOVA, ^*^P < 0.05, ^**^P < 0.01). Bacterial growth was conducted by injection 100 μL cell suspension (10^6^ CFU/mL) into the stem of four-week-old *N*. *benthamiana*. Plants were subjected to growth curve analysis at 3 dpi. Values are means ± SD (*n* = 6 biological replicates; Student’s *t*-test, ^*^P < 0.05, ^**^P < 0.01). **B**–**C** Virulence of Δ*ripAF1* mutant on *Solanum lycopersicum* (**B**) and *Capsicum annuum* (**C**) plants, characterized as in (**A**).

To examine the contribution of RipAF1 to *R. solanacearum* virulence, *Nicotiana benthamiana* plants were inoculated with wild-type FJ1003, the *ripAF1* deletion mutant Δ*ripAF1*, and the complemented strain CΔ*ripAF1* via stem wound inoculation. The mutant *ΔripAF1* caused more severe wilt symptoms relative to wild-type FJ1003. At 7 days post-inoculation, the stem near the inoculation site of plants infected with *ΔripAF1* was completely dark and shrunken (Fig. 2A). A disease index was used to monitor bacterial wilt development, and mutant *ΔripAF1* induced a higher disease index until 10 days post-inoculation (Fig. 2A). Accordingly, *ΔripAF1* grew faster than wild-type FJ1003 in *N. benthamiana* (Fig. 2A). The mutant *ΔripAF1* also showed increased disease severity, disease index, and replication speed in both *Solanum lycopersicum* and *Capsicum annuum* (Fig. 2B and 2C). To further validate the role of RipAF1 in virulence, a *ripAF1* deletion mutant generated from the GMI1000 strain was inoculated into *S. lycopersicum* and *C. annuum*. Compared with wild-type GMI1000, the *ripAF1* mutant caused more severe symptoms, increased disease indexes, and accelerated growth speeds in both *S. lycopersicum* and *C. annuum* plants (Fig. S2, see online supplementary material).

### ADP-ribosylation activity is required for RipAF1 functions

**Figure 3:**
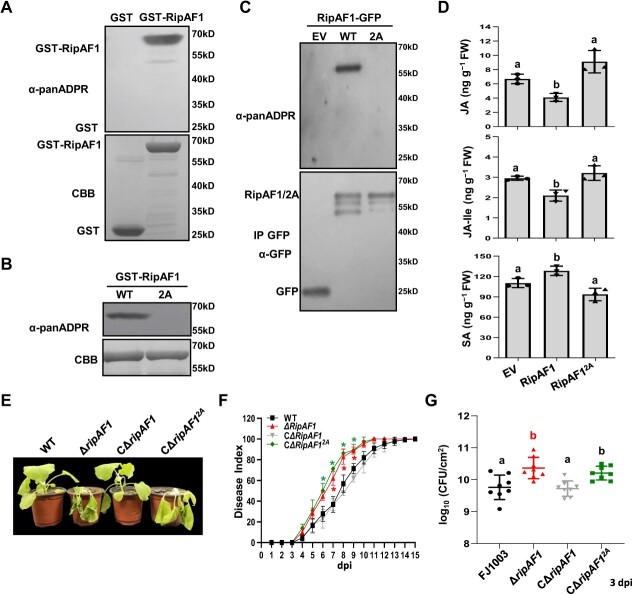
Assays for the ADP-ribosyltransferase activity of RipAF1. **A** RipAF1 displayed ADP-ribosyltransferase (ADP-RT) activity *in vitro*. The ADP-RT activity of recombinant GST-RipAF1 was examined using anti-panADPR reagent. GST served as the negative control. Protein abundance was revealed by Coomassie brilliant blue (CBB) staining. Similar results were obtained from three independent replicates. **B** Mutation of R191 and D310 in RipAF1 to alanine impaired ADP-RT activity. GST-RipAF1 and GST-RipAF1^2A^ (R191 arginine and D310 aspartic were substituted with alanine) were subjected to ADP-RT activity analysis. Protein abundance was indicated by CBB staining. Similar results were obtained from three independent replicates. **C** The loss of ADP-RT activity of RipAF1^2A^*in vivo*. RipAF1-GFP and RipAF1^2A^-GFP were expressed in *Nicotiana benthamiana,* immunoprecipitated with anti-GFP agarose beads and subjected to ADP-RT activity analysis. GFP was used as the negative control. The experiment was replicated three times with similar results. **D** Biosynthesis of JA, jasmonoyl-isoleucine (JA-Ile) and SA in *N*. *benthamiana* expressing RipAF1^2A^. Phytohormone levels were quantified by HPLC–MS/MS. Values are means ± SD (*n* = 3 biological replicates). Columns labeled with different letters indicate significant difference among means (ANOVA with Tukey’s test, *P* < 0.05). **E** Disease symptom of plants inoculated CΔ*ripAF1^2A^*. Images were captured 7 d post-inoculation. The experiments were replicated three times with similar results, and representative results are shown. **F** Disease index on *N*. *benthamiana* plants. Disease severity was recorded till 15 dpi. Each time point represents the mean disease severity of 24 inoculated plants per treatment. Error bars represent the standard deviation from three independent experiments (two-way ANOVA, ^*^P < 0.05). **G** Growth of CΔ*ripAF1^2A^* in *N*. *benthamiana* plants. The stem of four-week-old *N*. *benthamiana* was injected with 100 μL of 10^6^ CFU/mL bacterial cell suspension. Plants were subjected to growth curve analysis at 3 dpi. Labeling with different letters indicates significant differences between means. Values are means ± SD (*n* = 8 biological replicates; ANOVA Tukey’s test, *P* < 0.01).

As RipAF1 possesses an ADP-RT domain, an anti-pan-ADP-ribose binding reagent (anti-panADPR) was used to examine whether RipAF1 has ADP-RT activity. GST-tagged RipAF1 was first expressed in *Escherichia coli* strain BL21 and purified for subsequent analysis. The ADP-RT activity of RipAF1 was successfully detected by using anti-panADPR (Fig. 3A). The ADP-RT domain of RipAF1 showed 37% identity with that of HopF2, whose Arg71 and Asp175 residues are critical for its ADP-RT activity (Fig. S3A-D, see online supplementary material). A mutant RipAF1 was created by replacing Arg191 and Asp310 with alanine, thus generating RipAF1^R191A/D310A^ (RipAF1^2A^). The ADP-RT activity of RipAF1^2A^ was impaired when it was expressed in *E. coli* (Fig. 3B). Immunoprecipitation of RipAF1^2A^-GFP from *N. benthamiana* revealed that its ADP-RT activity was completely lost *in planta* (Fig. 3C). RipAF1^2A^ without ADP-RT activity had no effect on its secretion by *Rso* (Fig. S3E, see online supplementary material), meanwhile it showed the same subcellular localization as wild-type RipAF1. Fluorescence was observed in the cell membrane, cytoplasm, and nucleus when RipAF1^2A^-GFP was transiently expressed in *N. benthamiana* (Fig. S3F, see online supplementary material). However, RipAF1^2A^ lost the ability to suppress JA signaling marker genes *NbPDF1.2* and *NbLOX.* The suppression effect on *NbOPR3* was significantly attenuated (Fig. S4A and B, see online supplementary material). Simultaneously, the SA signaling marker genes were not induced by RipAF1^2A^ (Fig. S4C, see online supplementary material). Biosynthesis of JA, JA-Ile, SA in plants expressing RipAF1^2A^ showed no difference from those in control plants (Fig. 3D). The expression of RipAF1^2A^ in *ΔripAF1* had no distinctive effect on bacterial growth (Fig. S4D, see online supplementary material) and did not restore the virulence and replication phenotypes to those of wild-type FJ1003 (Fig. 3E–G).

### RipAF1 interacts with host FBN1

**Figure 4:**
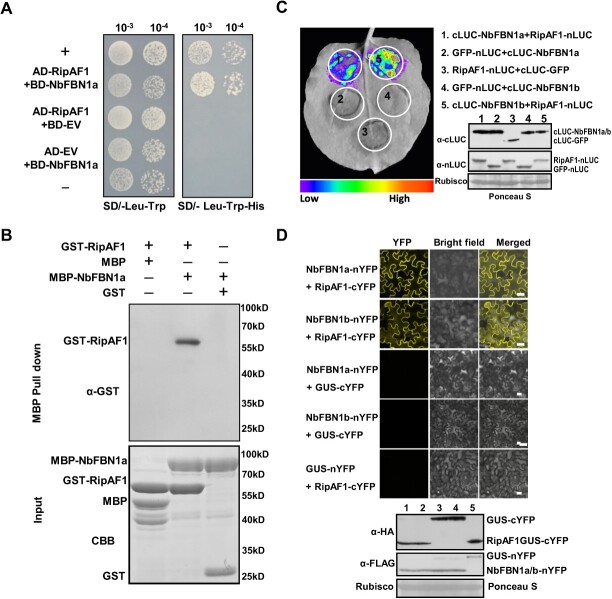
RipAF1 interactions with *Nicotiana benthamiana* FBN1 *in vitro* and *in vivo*. **A** Yeast two-hybrid analysis showing RipAF1 interacts with NbFBN1a. AD-RipAF1 and BD-NbFBN1a were co-transformed into yeast cells and screened on synthetic dextrose media lacking leucine and tryptophan (SD/−Leu-Trp). A single yeast colony was then selected for serial dilution and subsequent culture on SD/−Leu-Trp and SD/−Leu-Trp-His to examine the potential interaction. Yeast co-transformed with pGADT7-T and pGBKT7–53 served as the positive control (+), while yeast co-transformed with pGADT7-T and pGBKT7-lam served as the negative control (−). EV, empty vector. **B** MBP pull-down assay of the interaction between RipAF1 and NbFBN1a. The recombinant GST-RipAF1 and MBP-NbFBN1a were subjected to MBP pull-down analysis. MBP and GST tags served as the negative control. The pulled down GST-RipAF1 was detected by anti-GST immunoblotting. The experiment was replicated three times with similar results. **C** Split luciferase assay for the interaction of RipAF1 with NbFBN1a/b. *N. benthamiana* leaves were co-infiltrated with *Agrobacterium* carrying the 35S:RipAF1*-*nLUC and 35S:cLUC*-*NbFBN1a/b constructs. Images of chemiluminescence were obtained after application of 0.5 μM luciferin 48 h post-infiltration. Western blotting showed the expression of the respective proteins. Similar results were observed in three biological replicates. **D** Split YFP assay for the interaction of RipAF1 and NbFBN1a/b, performed as in (**C**). RipAF1 and NbFBNs were fused with cYFP and nYFP at the C terminus, respectively. The images were observed under a confocal microscope at 2 days post-agroinfiltration. Scale bar, 25 μm. Western blotting was used to confirm the expression of the respective proteins.

To identify the host protein that interacted with RipAF1, yeast two-hybrid (Y2H) assays were conducted using RipAF1 as bait to screen a *N. benthamiana* cDNA library. A positive clone was identified, and it harbored a 1162-bp DNA fragment corresponding to the fibrillin gene *FBN1a*. Except for a 966-bp opening read frame (ORF), the clone contained a 60-bp 5′ terminal untranslated region (UTR) and a 135-bp 3′ terminal UTR. The Y2H experiment was repeated, followed by cloning the *NbFBN1a* into the pGADT7 and pGBKT7 vectors. The positive interaction was verified after yeast AH109 was co-transformed with either pGADT7-RipAF1/pGBKT7-NbFBN1a or pGADT7-NbFBN1a/pGBKT7-RipAF1 (Fig. 4A; Fig. S5A, see online supplementary material). In a MBP pull down assay, MBP-NbFBN1a successfully pulled down GST-RipAF1. In the control lane, MBP tag did not pull down RipAF1, and the MBP-NbFBN1a construct was unable to pull down GST tag (Fig. 4B).

**Figure 5:**
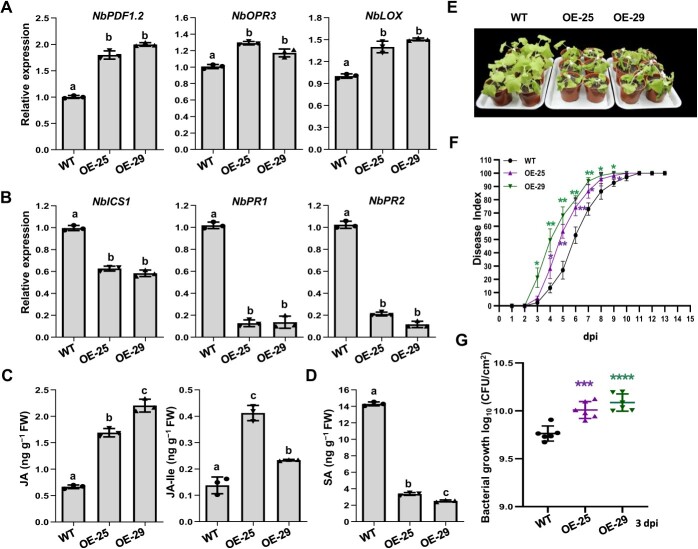
*NbFBN1a* transgenic *Nicotiana benthamiana* was more sensitive to bacterial wilt. **A** The expression levels of JA marker genes were enhanced in *NbFBN1a* transgenic plants. Total RNA was isolated from four-week-old transgenic *NbFBN1a*-overexpression lines OE-25 and OE-29. The transcript level for each gene in transgenic plants was compared with that in wild-type plants to monitor the expression change. Columns labeled with the same letter indicate means were not significantly different. Values are means ± SD (*n* = 3 biological replicates; ANOVA with Tukey’s test, *P* < 0.01). **B** The expression levels of SA marker genes were reduced in *NbFBN1a* transgenic plants, assayed as in (**A**). **C** and **D***NbFBN1a* transgenic plants showed increased contents of JA and JA-Ile (**C**) and reduced content of SA (**D**). Phytohormone levels were quantified by HPLC–MS/MS. Values are means ± SD (*n* = 3 biological replicates). Columns labeled with different letters indicate significantly different means (ANOVA with Tukey’s test, *P* < 0.01). **(E)** Wilt symptoms in wild-type and *NbFBN1a* transgenic plants inoculated with *Ralstonia solanacearum* FJ1003. Images were captured 7 d post-inoculation (dpi). All experiments were replicated three times with similar results, and representative results are shown. **F** Disease index of *NbFBN1a* transgenic plants inoculated with *R. solanacearum* FJ1003*.* Each time point represents the mean disease severity of 24 inoculated plants per treatment. Error bars represent the standard deviation from three independent experiments (ANOVA, ^*^P < 0.05, ^**^P < 0.01). **G** Growth of the FJ1003 strain in *NbFBN1a* transgenic plants. Plants stems were collected for growth curve analysis at 3 dpi with 100 μL of 10^6^ CFU/mL bacterial cell suspension. Values are means ± SD (*n* = 6 biological replicates; Student’s *t*-test, ^***^P < 0.001, ^****^P < 0.0001).

*Nicotiana benthamiana* has two copies of *FBN1*: *NbFBN1a* and *NbFBN1b* (*NbFBN1a/b* collectively hereafter)*.* Each of the inferred FBN1 proteins has 321 amino acids, sharing 95% identity with 16 amino acid differences (Fig. S5B, see online supplementary material). The extremely high identity suggests that NbFBN1a and NbFBN1b are functionally redundant in *N. benthamiana*. Fluorescence was mainly observed in the chloroplast when NbFBN1a/b-GFP was expressed in *N. benthamiana* (Fig. S5C, see online supplementary material). Transient expression assays using *N. benthamiana* protoplasts also indicated that NbFBN1a/b was mainly located in the chloroplast (Fig. S5D, see online supplementary material). Expression of RipAF1-mCherry in *N. benthamiana* revealed that RipAF1-mCherry was localized to the cytoplasm, membrane, and nucleus (Fig. S5E, see online supplementary material). Additionally, merged yellow fluorescence was observed in the plasma membrane when RipAF1-mCherry was transiently co-expressed with NbFBN1a/b-GFP (Fig. S5E, see online supplementary material). This demonstrated that RipAF1 and NbFBN1a/b were co-localized to the cell membrane. The interaction between RipAF1 and FBN1a/b *in vivo* was first verified by split luciferase assays (Fig. 4C). Then, the interactions at the plasma membrane were confirmed by BiFC assays (Fig. 4D). By using the membrane marker PIP2A-mCherry, merged orange fluorescence was localized to the cell membrane when membrane-anchored PIP2A-mCherry was co-expressed with BiFC constructs by plasmolysis treatment (Fig. S5F, see online supplementary material).

NbFBN1 homologs were found in both *S. lycopersicum* and *C. annuum.* Their amino acid sequences shared over 61% identity with NbFBN1a/b. In the phylogenetic analysis of FBN1 from *N. benthamiana*, *S. lycopersicum*, *C. annuum*, and *A. thaliana*, FBN1 from *S. lycopersicum* and *C. annuum* showed a close orthologous relationship with NbFBN1a/b (Fig. S6A, see online supplementary material). The interactions between RipAF1 and FBN1 from *S. lycopersicum* and *C. annuum* were verified by split luciferase assays (Fig. S6B, see online supplementary material).

### Overexpression of NbFBN1a increases JA signaling and suppresses SA signaling

To study the role of *NbFBN1* in bacterial wilt development, the NbFBN1a-GFP fusion construct was transiently overexpressed in *N. benthamiana*. The expression of NbFBN1a-GFP did not cause any visible phenotype. However, compared with the GFP control, NbFBN1a-GFP significantly induced the JA signaling pathway marker genes *NbPDF1.2*, *NbOPR3,* and *NbLOX* (Fig. S7A, see online supplementary material). In contrast, the expression levels of the SA signaling pathway marker genes *NbICS1*, *NbPR1*, and *NbPR2* were all suppressed (Fig. S7B, see online supplementary material). The NbFBN1a possesses a chloroplast transit peptide (cTP) domain from position 1 to 56 aa in the N-terminal region (Fig. S8A, see online supplementary material). The cTP^1–56^-GFP was found in chloroplasts when transiently expressed in *N*. *benthemiana*. The truncated NbFBN1a with deletion of cTP (NbFBN1a^57–321^) was located at the cell membrane (Fig. S8B, see online supplementary material). By comparison with expression of full length NbFBN1a, expression of NbFBN1a^57–321^ showed suppression effect on the expression of JA signaling marker genes, and induction effect on SA signaling marker genes (Fig. S8C and D, see online supplementary material). This suggested that membrane-localized FBN1a exerted a diverse role on SA and JA signaling.

To confirm the signaling pathways were altered, transgenic *N. benthamiana* plants expressing *NbFBN1a-GFP* were generated. Two independent homozygotes, lines 25 and 29, respectively, were chosen for further analysis after the overexpression of NbFBN1a was verified by qRT-PCR, western blot, and GFP fluorescence assays (Fig. S9A–C, see online supplementary material). In the two transgenic lines, the transcript levels of *NbPDF1.2*, *NbOPR3*, and *NbLOX* were enhanced (Fig. 5A), while the transcript levels of the SA signaling marker genes *NbICS1*, *NbPR1*, and *NbPR2* were reduced (Fig. 5B). In addition, the increased biosynthesis of JA and JA-Ile and the reduced biosynthesis of SA was quantified in transgenic plants (Fig. 5C and D). Transgenic plants were also examined for their resistance to bacterial wilt. The disease indexes of both transgenic lines were higher than that of wild-type plants, suggesting that lines 25 and 29 were more sensitive to *Rso* than was wild-type *N. benthamiana* (Fig. 5E and F). Consistent with the increased sensitivity observed, *Rso* FJ1003 replicated to a higher level in NbFBN1a-GFP transgenic plants (Fig. 5G).

### Knock-down of *NbFBN1* resulted in increased resistance to bacterial wilt

**Figure 6:**
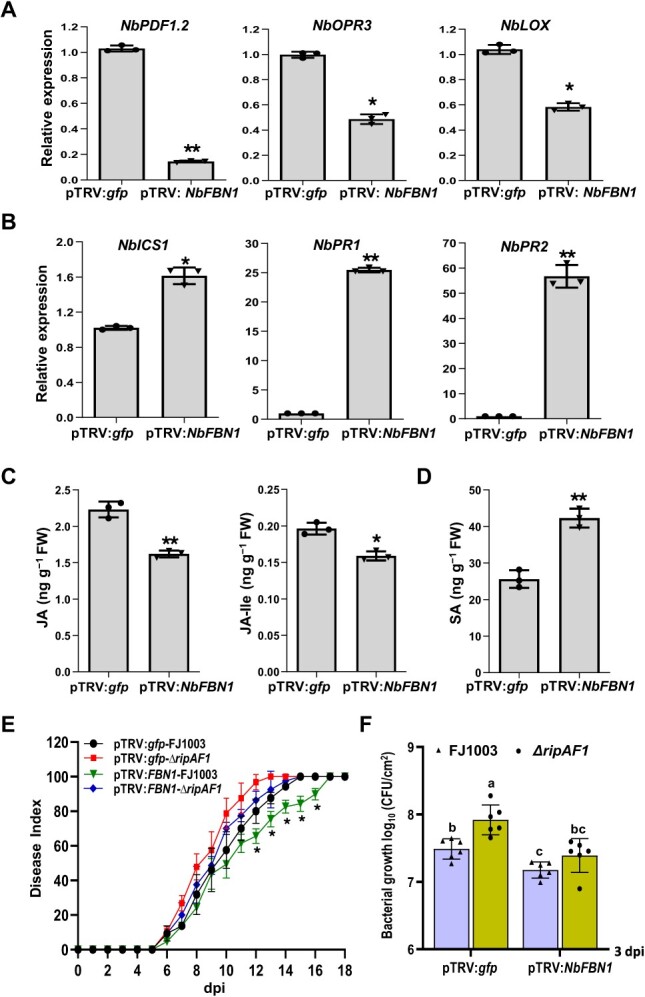
Knock-down of *NbFBN1* in *Nicotiana benthamiana* resulted in enhanced resistance to bacterial wilt. **A** Expression levels of JA signaling marker genes were reduced in pTRV:*NbFBN1* plants. Total RNA was isolated from the new upper leaves when photobleaching was observed in phytoene desaturase (*PDS*)-silenced plants (efficiency control). The transcript level in plants transformed with the tobacco rattle virus pTRV:*gfp* construct was used as a control to monitor expression change. Values are means ± SD (*n* = 3 biological replicates; Student’s *t*-test, ^*^P < 0.05, ^**^P < 0.01). **B** The expression levels of SA signaling marker genes were enhanced in pTRV:*NbFBN1* plants. The analyses were performed as in (**A**). **C** and **D** The reduced biosynthesis of JA and JA-Ile (**C**) and increased biosynthesis of SA (**D**) in pTRV:*NbFBN1* plants*.* Phytohormones were quantified by HPLC–MS/MS. Values are means ± SD (*n* = 3 biological replicates; Student’s *t*-test, ^*^P < 0.05, ^**^P < 0.01). **E** Disease index of pTRV:*NbFBN1* plants or pTRV:*gfp* inoculated with the *Ralstonia solanacearum* FJ1003 and Δ*ripAF1*. Each time point represents the mean disease severity of 24 inoculated plants per treatment. Error bars represent the standard deviation of three independent experiments (two-way ANOVA, ^*^P < 0.05). **F** Growth of *R. solanacearum* FJ1003 and ∆*ripAF1* in pTRV:*gfp* or pTRV:*NbFBN1* plants. The stems of plants were injected with 100 μL of 10^6^ CFU/mL bacterial cell suspension. Plants were subjected to growth curve analysis at 3 dpi. Error bars represent means ± standard deviation. Different letters above each column indicate a significant difference (two-way ANOVA, *P* < 0.05, *n* = 6).

The knock-down of *NbFBN1* in *N*. *benthamiana* was conducted by using tobacco rattle virus (TRV)-induced gene silencing. The pTRV:*gfp* construct carrying a 358-bp fragment of the *gfp* gene was used as a control. Compared with the *gfp-*silenced control, the transcript level of *NbFBN1* was decreased in pTRV:*NbFBN1* plants, indicating *NbFBN1a/b* was successfully knocked down (Fig. S10A and B, see online supplementary material). The knock-down of *NbFBN1* affected plant growth, especially root development (Fig. S10C, see online supplementary material). In the *NbFBN1*-silenced plants, the transcript levels of the JA marker genes *NbPDF1.2*, *NbOPR3*, and *NbLOX* were reduced (Fig. 6A). The expression levels of three SA signaling marker genes, *NbICS1*, *NbPR1*, and *NbPR2*, were all enhanced (Fig. 6B). Accordingly, JA and JA-Ile levels were significantly decreased and SA level was increased in *NbFBN1*-silenced plants (Fig. 6C and D). The inoculation of the FJ1003 strain into *NbFBN1*-silenced plants induced retarded disease development. The disease index was lower than that of pTRV:*gfp* control plants at 12, 13, 14, 15, and 16 days post inoculation (Fig. 6E). The disease development of mutant *ΔripAF1* plants showed no difference between pTRV:*gfp* and *NbFBN1-*silenced plants (Fig. 6E). The replication of either FJ1003 or ∆*ripAF1* was decreased in *NbFBN1-*silenced plants as compared to in pTRV:*gfp* plants. Additionally, a significant difference in bacterial growth was observed between the wild type and ∆*ripAF1* in pTRV: *gfp* plants. In contrast, replication of FJ1003 and ∆*ripAF1* showed no distinctive difference in *NbFBN1-*silenced plants (Fig. 6F). Knock-down of *NbFBN1* in *N*. *benthamiana* led to increased resistance to bacterial wilt.

### ADP-ribosylation of NbFBN1a is critical for JA and SA signaling modulation

**Figure 7:**
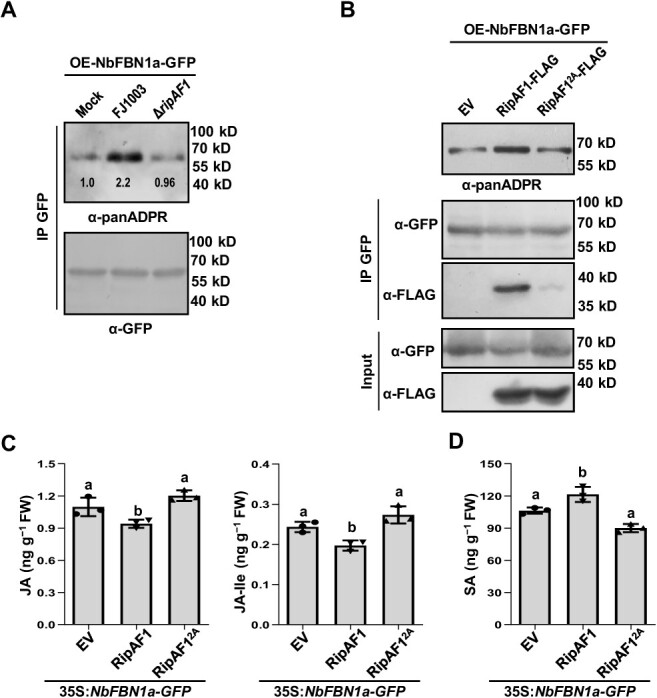
RipAF1 eliminates NbFBN1a-mediated JA and SA signaling dependent on ADP-ribosylation activity. **A** Δ*ripAF1* reduced the ADP-ribosylation level of NbFBN1a *in vivo*. Four-week-old *NbFBN1a-GFP* transgenic plant leaves were inoculated with FJ1003 or Δ*ripAF1* at a concentration of 3 × 10^7^ CFU/mL. Infected leaves were sampled for immunoblotting at 24 h post inoculation (hpi). Protein extracts were subjected to immunoprecipitation (IP) with anti-GFP agarose beads. The ADP-ribosylation level of NbFBN1a was detected with anti-panADPR antibody. Similar results were obtained from three biological replicates. The mock sample is the protein extracted from leaves infiltrated with 10 mM MgCl_2_. **B** NbFBN1a is ADP-ribosylated by RipAF1 *in vivo*. RipAF1-FLAG or RipAF1^2A^-FLAG was expressed in four-week-old *NbFBN1a* transgenic plant leaves. Protein extracts were subjected to immunoprecipitation (IP) with anti-GFP agarose beads and immunoblotting (IB) with anti-FLAG antibody. The ADP-ribosylation level of NbFBN1a was detected with anti-panADPR antibody. The experiment was replicated three times with similar results. **C** and **D** Biosynthesis of JA or JA-Ile (**C**) and SA (**D**) in *NbFBN1a-GFP* transgenic plant expressing RipAF1^2A^. Phytohormone levels were quantified by HPLC–MS/MS. Values are means ± SD (*n* = 3 biological replicates). Columns labeled with different letters indicate significantly different means (ANOVA with Tukey’s test, *P* < 0.05).

The ADP-ribosylation of NbFBN1a was assessed in NbFBN1a-GFP transgenic plants infected with *Rso*. Compared with wild-type FJ1003, *ΔripAF1* significantly reduced the ADP-ribosylation level of NbFBN1a (Fig. 7A). This suggested that the ADP-ribosylation level of NbFBN1a under *Rso* infection is dependent on the presence of RipAF1. The ADP-ribosylation of NbFBN1a was studied further by expressing RipAF1-FLAG in NbFBN1a-GFP transgenic plants. The anti-panADPR immunoblot revealed a positive band after NbFBN1a-GFP fusion protein was immunoprecipitated by GFP beads. Compared with wild-type RipAF1, expression of RipAF1^2A^ in transgenic plants greatly reduced the ADP-ribosylation level of NbFBN1a (Fig. 7B). In the *in vitro* ADP-ribosylation experiment, when GST-RipAF1 and MBP-NbFBN1a are co-incubated, the ADP-ribosylation level of NbFBN1a can be detected. However, no band is detected when it incubated with GST alone. This suggests that RipAF1 can ADP-ribosylate NbFBN1a *in vitro*. (Fig. S11A, see online supplementary material). Compared with wild-type RipAF1, expression of RipAF1^2A^ in transgenic plants did not suppress the biosynthesis of JA or induce the biosynthesis of SA (Fig. 7C and D, see online supplementary material). This demonstrated that RipAF1^2A^ was unable to eliminate NbFBN1a-mediated JA and SA signaling.

### E175/K207 are the major residues enabling NbFBN1a to be ADP-ribosylated by RipAF1

**Figure 8:**
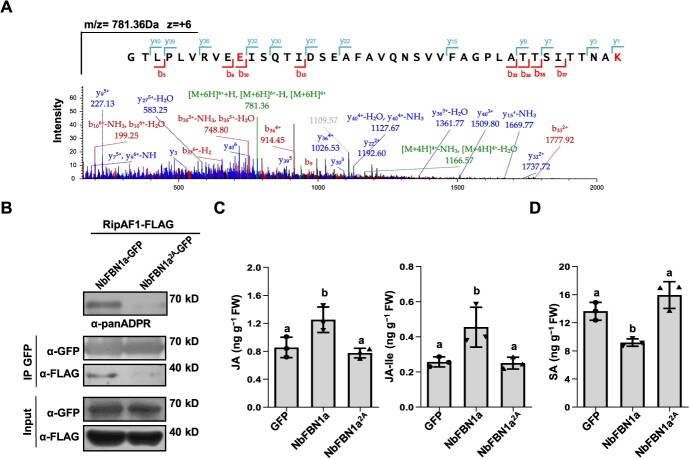
The major ADP-ribosylation sites of NbFBN1a. **A** LC–MS/MS analysis of a peptide containing NbFBN1a with ADP-ribosylated E175/K207 residues. Recombinant GST-RipAF1 and His-MBP-NbFBN1a proteins were co-expressed in *E. coli*. Trypsin-digested recombinant proteins were subjected to LC–MS/MS analysis. Fragment ions retaining charges in the N terminus and C terminus are denoted by ‘b’ and ‘y’, respectively. **B** The E175/K207 residues of NbFBN1a were essential for effective ADP-ribosylation by RipAF1. RipAF1-FLAG was co-expressed with NbFBN1a-GFP or NbFBN1a^2A^-GFP in wild-type *N*. *benthamiana*. In NbFBN1a^2A^, the glutamic acid at position 175 (E175) and lysine at position 207 (K207) were both substituted with alanine. Protein extracts were subjected to IP with GFP resins and IB with anti-FLAG antibody. Input proteins are shown by anti-FLAG IB and Ponceau S staining for protein loading (bottom). The ADP-ribosylation levels of NbFBN1a and NbFBN1a^2A^ were detected by anti-panADPR antibody. The experiment was replicated three times, with similar results. **C** and **D** Biosynthesis of JA and JA-Ile (**C**) and SA (**D**) in *N*. *benthamiana* expressing NbFBN1a^2A^-GFP*.* Phytohormone levels were quantified by HPLC–MS/MS. Values are means ± SD (*n* = 3 biological replicates). Columns labeled with different letter indicate significantly different means (ANOVA with Tukey’s test, *P* < 0.01).

To determine the putative ADP-ribosylation acceptor sites in NbFBN1a modified by RipAF1, His-MBP-tagged NbFBN1a was co-expressed with GST-RipAF1 in *E. coli*. The purified His-MBP-tagged NbFBN1a was subjected to liquid chromatography-mass spectrometry (LC–MS/MS) analysis followed by higher-energy collision dissociation in order to identify the characteristic marker ions corresponding to adenine, adenosine-H_2_O, adenosine monophosphate (AMP), adenosine diphosphate (ADP), and ADP-ribose. By comparison with the co-expressed GST-RipAF1^2A^ control, 19 positions of NbFBN1a were found to be ADP-ribosylated, including six glutamic acid sites (E71, E84, E140, E175, E235, E297) and four lysine sites (K50, K148, K207, K313) (Table S1, see online supplementary material). The E175 and K207 positions (E175/K207) were revealed from the most abundant peptide fragment corresponding to residues 166–207 of NbFBN1a (Fig. 8A; Fig. S11B, see online supplementary material). E175/K207 residues are highly conserved among FBN1 from *S. lycopersicum*, *S. tuberosum*, *C. annuum*, *A. thaliana*, and *O. sativa* (Fig. S11C, see online supplementary material). To elucidate the roles of E175/K207 residues in ADP-ribosylation, they were mutated to alanine, thus generating NbFBN1a^E175A/K207A^ (NbFBN1a^2A^). In this case, the ADP-ribosylation level of NbFBN1a^2A^ was significantly reduced relative to wild-type NbFBN1a *in vivo* (Fig. 8B). This implied that E175/K207 residues in NbFBN1a are critical for ADP-ribosylation by RipAF1.

To examine the potential role of the ADP-ribosylation process for NbFBN1a-mediated signaling, NbFBN1a^2A^ was transiently overexpressed in *N. benthamiana.* In contrast to NbFBN1a, NbFBN1a^2A^ significantly reduced the ability to activate the JA signaling marker genes *NbPDF1.2*, *NbOPR3,* and *NbLOX* and inhibited the expression of the SA signaling marker genes *NbICS1*, *NbPR1*, and *NbPR2* (Fig. S11D and E, see online supplementary material). Simultaneously, biosynthesis of JA, JA-Ile, and SA showed no difference from control plants (Fig. 8C and D, see online supplementary material). Two synthetic *NbFBN1a-S* and *NbFBN1a^2A^-S* sequences were constructed within which the nucleotide sequences were mutated, enabling their expression to be unaffected by gene silencing (Fig. S12A and B, see online supplementary material). After being fused with GFP in pC1300s vector, the two constructs were transiently overexpressed in TRV:*NbFBN1* plants. NbFBN1a^2A^-S showed significantly reduced ability to activate the promoter activity of *NbPDF1.2* relative to NbFBN1a-S (Fig. S12C, see online supplementary material)*.* This suggests that the activation of JA signaling by NbFBN1a was dependent on the ADP-ribosylation level of NbFBN1a.

## Discussion

The present study was the first to report effector-mediated ADP-ribosylation in the *Rso*–host interaction system. This modification mode has been well documented in plant-associated *P. syringae* effectors. In addition to the known HopF2 protein, HopU1 modifies RNA-binding proteins, including the glycine-rich RNA-binding protein GRP7, to suppress plant innate immunity in a manner dependent on its ADP-RT active residue [35]. *Rso* RipAF1 contains a conserved ADP-RT domain at positions 155–338 (Fig. S1D, see online supplementary material), which shows high identity with the ADP-ribosylation activity domain of HopF2. Relying on the active ADP-ribosylation activity on the host target, RipAF1 modulates antagonistic JA and SA signaling for bacterial wilt development.

*Rso* promotes disease severity by inducing JA signaling and suppressing SA signaling in plants [7]. The plant hormones JA and SA are well-known signaling compounds involved in the regulation of plant defense [6]. In the *Rso* strain RS1002, RipAL plays a key role in inducing JA production to activate JA signaling and simultaneously to suppress SA-mediated defense responses in susceptible *C. annuum* plants, contributing to severe disease development [7]. To overcome host defense responses, *Rso* employs a set of effectors to suppress host innate immunity. Deletion of *Rip*-encoding genes usually leads to reduced virulence in host plants. Previous work reported that RipAF1 from RS1000 shows no distinct effect on suppressing flg22-triggered innate immunity [7]. Overexpression of RipAF1 in *N. benthamiana* reduced the biosynthesis of JA and JA-Ile, as well as the increased biosynthesis of SA (Fig. 1). This may explain why deletion of RipAF1 in *Rso* resulted in increased disease severity in hosts. The enhanced virulence of Δ*ripAF1* (Fig. 2) demonstrated a particular role of this effector in *Rso*-host interaction.

A prominent trait of the *Rso* species complex is that it infects a wide range of hosts, with host specificity and/or fitness that is determined by the repertoire of Rips [13, 36–39]. RipP1 and RipAA from GMI1000 and RipB from RS1000 induce hypersensitive response (HR) reactions and are involved in incompatible interactions in *Nicotiana* spp. [40, 41]. Deletion of RipAX2 in GMI1000 converts an incompatible interaction to a compatible one in resistant AG91–25 eggplant carrying *R* genes at the *ERs1*/*EBWR9* locus [42]. Even though mutagenesis was conducted in both FJ1003 and GMI1000 strains, we did not find a contribution of RipAF1 to host specificity in *N. benthamiana*, *C. annuum*, or *S. lycopersicum*.

The RipAF1-GFP fusion construct in this study was observed in the cytoplasm, cell membrane, and nucleus, which is consistent with a previous report [16]. *Arabidopsis* AtFBN1a was found to be located in the chloroplast [43]. Similarly, NbFBN1a was observed in the chloroplast of *N. benthemiana* (Fig. S5C and D, see online supplementary material). Furthermore, RipAF1 and NbFBN1a interacted at the cell membrane *in vivo* (Fig. 4). As in *A. thaliana* [44], there are two copies of NbFBN1 in *N. benthamiana*, namely NbFBN1a and NbFBN1b, which share high similarity with homologs from various solanaceous plants (Fig. S5B, see online supplementary material). The FBNs are widely distributed among photosynthetic organisms [45]. In addition to its interactions with NbFBN1a/b, RipAF1 also interacts with NbFBN1 homologs from *S. lycopersicum* and *C. annuum*, implying that FBN1 homologs in the two hosts are also targeted by RipAF1 (Fig. S6, see online supplementary material).

FBN1 has been well documented for its role in plastoglobule formation and thylakoid maintenance, especially *Arabidopsis* AtFBN1a and AtFBN1b [44, 46, 47]. Knock-out of *FBN1b* in *Arabidopsis* and knock-down of *FBN1* in *L. esculentum* resulted in increased susceptibility to *P. syringae* and *B. cinerea*, respectively [31]. Through overexpression and RNA interference analysis, *NbFBN1a* was demonstrated to be involved in JA and SA signaling pathways (Figs 5 and 6). In contrast with the resistance roles of homologs in *Arabidopsis* and *L. esculentum*, NbFBN1a in *N. benthamiana* plays a role in susceptibility to *Rso*. The *N. benthamiana* plants overexpressing NbFBN1a exhibited more severe bacterial wilting symptoms, while knock-down of NbFBN1a/b enhanced resistance to bacterial wilt. This supported the hypothesis that host FBN1 plays an opposing role in resistance against various host-pathogen interactions.

In agreement with past research on *P. syringae* pv. *tomato* HopF2 [18], the conserved Arg191 and Asp310 residues were critical for RipAF1 ADP-RT activityFig. S5A–D, see online supplementary materialFig. 3. By combining LC–MS/MS and site-directed mutagenesis analysis, it was found that RipAF1 directly ADP-ribosylates NbFBN1a. Based on LC-MS/MS analysis, 19 putative ADP-ribosylation acceptor sites were identified from NbFBN1a. Other methods are needed to carefully verify ADP-ribosylation on those sites. Here we examined the requirement of conserved E175/K207 residues for ADP-ribosylation. The generated mutant NbFBN1a^2A^ was impaired in its ability to modulate associated signaling. ADP-ribosylation of NbFBN1 resulted in inhibition of JA signaling and activation of SA signaling pathways in host plants (Figs 7 and 8). Thus, it is proposed that ADP-ribosylation of NbFBN1a is the key process by which RipAF1 induces a resistance reaction. It must be mentioned that NbFBN1a can be naturally ADP-ribosylated in *N. benthamiana*. However, upon *Rso* infection, the presence of RipAF1 increased the ADP-ribosylation level.

In contrast with HopF1 and HopF2, which are required for virulence in *P. syringae* [18], mutation of RipAF1 enhanced virulence of *Rso*. We suggested that this difference is explained by the different targets during bacterial infection. HopF1 is recognized by the *R1* gene in soybean to determine the incompatible interaction [17]. Expression of HopF2 in *P. syringae* pv. *tabaci* induced a HR reaction in tobacco W38 plants [18]. Moreover, HopF2 targets MKK5, RIN4, and BAK1 to suppress plant innate immunity [20, 21]. Our data showed that overexpression of RipAF1 in *N. benthamiana* did not induce an obvious HR phenotype, while RipAF1 activated the plant resistance reaction by ADP-ribosylation of NbFBN1a. Along with the alteration in signaling pathways, *NbFBN1-*silenced plants expressed a dwarf phenotype with decreased root length. This implied that NbFBN1a/b is required for the growth of *N. benthamiana*, showing a function similar to that of AtFBN1a in *Arabidopsis* [24].

Overall, these data confirm that *Rso* RipAF1 negatively contributes to virulence. It modulates the antagonistic interaction between JA and SA signaling pathways relying on its ADP-ribosylation activity. Specifically, it targets and directly ADP-ribosylates host FBN1 to modulate JA and SA signaling for bacterial wilt development.

## Materials and methods

### Plant and bacterial materials

Seeds of *N. benthamiana*, *S. lycopersicum* cv. ‘Hongyangli,’ and *C. annuum* cv. ‘Yanshan01’ were obtained from the plant bacterium research group at Fujian Agriculture and Forestry University in Fujian Province, China (Fujian Province, China). Seeds were germinated and the resulting seedlings were grown in a greenhouse under a 16/8-hour light/dark photoperiod at 25°C. *Rso* FJ1003 and GMI1000 strains were used for virulence assays. The *ripAF1* mutants derived from FJ1003 and GMI1000 and the corresponding complementary strains were used to examine the contribution of RipAF1 to virulence. The complementary strains were generated by cloning *ripAF1* and its native promoter into the plasmid pBBR1MCS-5. Site-directed mutagenesis of RipAF1 to obtain RipAF1^2A^ (Arg191 and Asp310 residues were replaced with alanine) was conducted by overlap extension PCR with the primers RipAF1-R191A-F/R and RipAF1-D310A-F/R (Table S2, see online supplementary material). The constructs were verified by sequencing and then introduced into the *ΔripAF1* mutant strain by using electroporation.

### *R. solanacearum* infection assay

Bacterial virulence and plant resistance were evaluated based on disease severity and the percentage severity index. *Rso* cells were prepared to a concentration of 1 × 10^8^ CFU/mL. Wounded stems of four-week-old plants were then inoculated with the bacterial suspension. [48]. Inoculated plants were maintained at 28°C for the calculation of disease severity and percentage severity index. The percent severity indexes were recorded as previously described [48]. All experiments were replicated three times, and each replicate contained eight plants for every strain. Bacterial growth *in planta* was determined as described previously [48]. In brief, 100 μL of 1 × 10^6^ CFU/mL bacterial suspension was injected into four-week-old plant stems. The stem samples were collected from between 1 cm above and below the injection sites 3 days post-inoculation (dpi). Bacterial growth was quantified as the number of CFU per gram of stem. The experiment was conducted with three independent biological replicates.

### Yeast two-hybrid assays

Yeast two-hybrid assays were conducted to screen for RipAF1-interacting proteins within the cDNA library of *N. benthamiana* according to a standard LiAc-mediated transformation kit (Matchmaker; Clontech, Mountain View, CA, USA). To investigate the interaction, the coding sequences of *RipAF1* and *NbFBN1a* were cloned individually into both pGADT7 and pGBKT7 vectors. AD-RipAF1/BD-NbFBN1a or AD-NbFBN1a/BD-RipAF1 constructs were then transformed into yeast strain AH109 and screened on synthetic drop-out media lacking leucine and tryptophan (SD/−Leu-Trp). Single colonies were cultured, serially diluted, and grown on SD/−Leu-Trp and SD/−Leu-Trp-His media to examine the potential interaction. Subsequently, 1 mM 3-amino-1,2,4-triazole (3-AT) was added to SD/−Leu-Trp-His medium to prevent the autoactivation of AD-NbFBN1a when AD-NbFBN1a/BD-RipAF1 was co-transformed into yeast. Positive controls included pGADT7-T and pGBKT7–53, while negative controls consisted of pGADT7-T and pGBKT7-lam.

### MBP pull-down assay

The *NbFBN1a* gene was inserted into the pMAL-C4X vector to enable the expression of the MBP-NbFBN1a fusion protein. Similarly, the *RipAF1* gene was cloned into the pGEX-4 T vector to express the GST-RipAF1 fusion protein. The expression of MBP-NbFBN1a and GST-RipAF1 was induced separately in *E. coli* BL21(DE3) by the induction of 1.0 mM isopropyl-β-D-thiogalactopyranoside (IPTG). Following purification using glutathione resin and amylose affinity chromatography, MBP pull-down assays were conducted [12].

### Western blotting analysis

Western blot analysis was conducted to evaluate the protein expression in *N*. *benthamiana* using a previously established technique [49]. Leaf samples were harvested at 48 hpi. The plant leaves were pulverized in liquid nitrogen, and then extracted using extraction/washing buffer (Roche). The eluted proteins were mixed with 1.5× Laemmli loading buffer, separated by 10% (*w*/*v*) SDS-PAGE, and analysed through western blotting utilizing anti-FLAG (Abmart, Shanghai, China) or anti-GFP polyclonal antibody (Abmart).

RipAF1-FLAG and RipAF1^2A^-FLAG fusion constructs were expressed in cosmid pBBRMCS-1 and transformed into the mutant Δ*ripAF1* derived from FJ1003. The Δ*ripAF1* mutants carrying each construct were grown in 5 mL of NB medium overnight at 28°C, harvested by centrifugation, and washed twice with M63 medium. The cell pellets were then inoculated into 150 mL of M63 liquid medium and cultured until OD_600_ reached 1.5. Proteins from cells and supernatant fractions were prepared as described previously [50]. The protein samples were then subjected to immunoblot analysis using anti-FLAG (Abmart).

### Luciferase assay and detection

The split-luciferase complementation assay was performed to verify the interaction between RipAF1 and host FBN1 *in vivo*. *FBN1* genes from *N. benthamiana*, *S. lycopersicum*, and *C. annuum* were fused individually with cLUC at the C terminus. Subsequently, each cLUC-FBN1 construct was co-expressed with nLUC-RipAF1 in four-week-old *N. benthamiana* leaves. To examine the role of conserved residues involved in the putative interaction, RipAF1^2A^ was fused with nLUC to generate nLUC-RipAF1^2A^. NbFBN1a^2A^ (with E175/K207 residues each replaced by alanine) was fused with cLUC by overlap extension PCR with the primers NbFBN1a-E175A-F/R and NbFBN1a-K207A-F/R (Table S2, see online supplementary material) to generate cLUC-NbFBN1a^2A^. For *PDF1.2* promoter activity analysis, a 1493-bp promoter region in the pDONR207 vector was directly introduced into the destination vectors pGWB435 via Gateway cloning to generate pro:*PDF1.2*-LUC. The activity of the *PDF1.2* promoter was observed following transient expression in *N. benthamiana.* Leaves were collected at 48 hpi, treated with 0.5 mM luciferin, and dark-adapted for 1 minute to quench fluorescence. Luciferase luminescence images were captured using a cooled CCD imaging system (Roper Scientific, Trenton, NJ, USA). The strength of the luciferase signal was quantified to assess both interaction affinity and promoter activity. Briefly, leaf discs were treated with 100 μL of water containing 0.5 mM luciferin in a 96-well plate, and luminescence was measured using a microplate luminescence reader (Varioskan Flash; Thermo Scientific, Waltham, MA, USA).

### Subcellular localization analysis

Subcellular localization of every fluorescence fusion construct was examined with a confocal laser microscope (Leica Model TCS SP8; Leica, Wetzlar, Germany) at 48 h post-transient expression in *N. benthamiana.* RipAF1-GFP and NbFBN1a/b-GFP were observed at a 488 nm excitation wavelength with a 470–550 nm bandpass emission filter. The subcellular localization of NbFBN1a/b-GFP was additionally examined in the protoplast of *N. benthamiana*. For co-localization analysis, RipAF1 was cloned into pGD-3G-mCherry, generating a RipAF1-RFP fusion construct. RipAF1-RFP was co-expressed with GFP-NbFBN1a/b and observed at a 514 nm excitation wavelength with a 530–560 nm bandpass emission filter. Both pSCYNE and pSCYCE plasmids were used for the BiFC assays. RipAF1 was cloned into pSCYCE at the N terminus, generating the RipAF1-cYFP construct. NbFBN1a/b sequences were cloned into pSCYNE at the N terminus, generating NbFBN1a/b-nYFP constructs. RipAF1^2A^-cYFP and NbFBN1a^2A^-nYFP were additionally constructed for BiFC assays. Plasmolysis was performed by submerging the samples in 10 mM NaCl for 5 min before examining the subcellular localization. Yellow fluorescence was observed at a 514 nm excitation wavelength with a 500–550 nm band-pass emission filter. The membrane-bound marker PIP2A-mCherry was applied in the BiFC assay to verify membrane localization. Every subcellular localization analysis was replicated three times.

### Generation of NbFBN1a-GFP transgenic plants

To generate *NbFBN1a-GFP* overexpressing plants, the *NbFBN1a* coding sequence was cloned into the pCAMBIA1300s-GFP binary vector driven by a CaMV 35S promoter. The construct was verified by sequencing and then used for plant transformation via the leaf-disc method after introduction into *Agrobacterium tumefaciens* strain HB101. These transformations were performed by Wuhan BioRun Biosciences Co., Ltd (Wuhan, China). The *NbFBN1a-GFP* transgenic plants were selected with 25 μg mL^−1^ hygromycin. The primer pair NbFBN1a-GFP-F and NbFBN1a-GFP-R was used for PCR amplification to verify transgene integration in T0 and T1 offspring. T2 transgenic homozygous lines were used for gene expression and disease resistance analysis. The expression of NbFBN1a-GFP was verified by western blot with α-GFP antibody.

### Phytohormone measurement

Phytohormones were quantified by Nanjing Convinced-Test Technology Company (Nanjing, China) using HPLC-MS/MS. Leaves (1.5g in fresh weight) were homogenized in liquid nitrogen and extracted twice at 4°C for 1 h using an extraction solvent of isopropanol-water-hydrochloric acid (2:1:0.002, *V*:*V*:*V*). The extracted samples were spiked with H_2_JA (Tokyo Chemical Industry, Tokyo, Japan) and d4-SA (OlchemIm, Olomouc, Czech Republic) as internal standards. Phytohormones were then measured with an Agilent 1290 HPLC device (Agilent Technologies, Santa Clara, CA, USA) with an AB SCIEX QTRAP 6500 MS/MS system (AB SCIEX, Framingham, MA, USA). Experiments were performed with three independent biological replicates.

### Virus-induced gene silencing

A 300-bp partial sequence of *NbFBN1a* was cloned into the pTRV2 vector to knock down *NbFBN1a/b* in *N. benthamiana*; the pTRV2:*NbPDS* construct served as the control to assess silencing efficiency. The pTRV2:*gfp* construct, carrying a 358-bp fragment of the *gfp* gene, was used as the negative control [49]. A mixture of GV3101 cultures (1:1, *v*/*v*) containing pTRV1 and pTRV2 constructs was co-infiltrated into 20-day-old *N*. *benthamiana* leaves. When *NbPDS*-silenced plants showed a photo-bleaching phenotype, qRT-PCR was performed to detect the *NbFBN1a/b* transcript levels. *NbFBN1a/b*-silenced plants were evaluated for signaling marker gene expression, phytohormone contents, and bacterial wilt resistance.

### Co-immunoprecipitation

Co-immunoprecipitation (Co-IP) was performed on homozygous *NbFBN1a-GFP* transgenic plants according to a previously described method [13]. At 48 hpi transient expression of RipAF1-FLAG in transgenic plants, 1.5-g samples of inoculated leaves were ground in liquid nitrogen and extracted in extraction/washing buffer containing 150 mM NaCl, 50 mM Tris–HCl (pH 7.5), 1 mM DTT, 0.2% Nonidet P-40, 0.1% Triton-X100, 1 × complete protease inhibitor (Roche, Shanghai, China), and 1 × phosphatase inhibitor tablet (Roche). The homogenate was centrifuged at 12000 rpm for 30 min. GFP-Trap_A (ChromoTek, Planegg, Germany) was introduced into the supernatant. After incubating at 4°C with end-over-end shaking for 2 hours, the beads were centrifuged at 2000 rpm for 2 minutes and then washed with washing buffer at least six times. The proteins bound to the beads were eluted using 1.5× Laemmli loading buffer, separated by 10% SDS-PAGE, and subsequently analysed through western blotting.

### LC–MS/MS analysis of ADP-ribosylation residues

To identify the residues of NbFBN1a ADP-ribosylated by RipAF1, GST-RipAF1 and MBP-NbFBN1a were co-expressed in *E. coli* strain BL21. MBP-NbFBN1a was purified by amylose affinity chromatography. Ten micrograms of MBP-NbFBN1a protein were incubated with 3 × Laemmli loading buffer at 100°C for 5 min. The denatured proteins were separated by 8% SDS-PAGE and then subjected to trypsin digestion. ADP-ribose peptides were analysed by the Q Exactive LC–MS/MS system (Thermo Scientific, Beijing Qinglian Biotech Co., Ltd, Beijing, China) [51, 52]. Acquired mass spectra were analysed using MaxQuant software, and the ADP-ribosylation sites were manually verified including marker ions at m/z 136.0632, 250.0940, 348.0709, and 428.0372.

### Detection of ADP-ribosylation activity

An anti-panADPR [52, 53] (catalog no. MABE1016, EMD Millipore, Burlington, MA, USA) was used to detect ADP-ribosylated proteins. GST-RipAF1 or GST-RipAF1^2A^ was expressed in *E. coli* strain BL21(DE3) and purified with a glutathione resin. To detect the ADP-ribosylation of RipAF1 *in vivo*, RipAF1-GFP and RipAF1^2A^-GFP were transiently expressed in *N. benthamiana* plants and purified by using GFP-Trap A (ChromoTek, Planegg, Germany). The bound proteins were eluted with 3 × Laemmli loading buffer, resolved by 10% SDS-PAGE, and then subjected to ADP-ribosylation analysis using a 1:5000 (*v*/*v*) dilution of anti-panADPR.

To detect ADP-ribosylated NbFBN1a, RipAF1-FLAG was first transiently expressed in *NbFBN1a-GFP* transgenic plants. The expression of RipAF1^2A^-FLAG was used as a negative control. NbFBN1a-GFP proteins were pulled down by Co-IP using GFP-Trap_A (ChromoTek) for ADP-ribosylation analysis. To verify the involvement of E175/K207 residues in ADP-ribosylation, RipAF1-FLAG and NbFBN1a^2A^-GFP were transiently co-expressed in *N. benthamiana* plants. The co-expression of RipAF1-FLAG and NbFBN1a-GFP was used as the positive control. The ribosylated NbFBN1a^2A^-GFP and NbFBN1a-GFP were detected by the anti-pan-ADP-ribose binding reagent after they were pulled down with Co-IP by GFP-Trap_A (ChromoTek). An *in vitro* ADP-ribosylation assay was determined as described previously [18]. In brief, these GST-RipAF1 and MBP-NbFBN1a were mixed in an ADPRT reaction buffer (40 mM HEPES, pH 7.5, 1 mM DTT, 60 mM ATP, 5 mM MgCl_2_, and 30 mM biotin-NAD^+^) for the reaction. GST was co-incubated with MBP-NbFBN1a, serving as a negative control. The reaction was terminated by boiling in 3 × Laemmli loading buffer, followed by analysis using 10% SDS-PAGE. The level of protein ADP-ribosylation was detected with anti-panADPR antibody.

### Sequence analysis

The distribution of RipAF1 in diverse phylotypic *Rso* strains was collected from the Ralsto T3E database (https://iant.toulouse.inra.fr/bacteria/annotation/site/prj/T3Ev3/). The RipAF1 and fibrillin sequences were downloaded from the UniProt database (http://uniprot.org). Sequence alignments were generated using Clustal X with default settings and analyzed by Espript3.0 (http://espript.ibcp.fr/ESPript/cgi-bin/ESPript.cgi). The phylogenetic tree was constructed from amino acid sequences utilizing MEGA 7.0. The evolutionary history of the analysed taxa was represented by the bootstrap consensus tree generated from 1000 replicates. The consensus amino acid positions of *Rso* RipAF1 and host FBN1 were generated using WebLogo (http://weblogo.threeplusone.com/create.cgi). Three-dimensional structures were predicted by Rosetta and RaptorX (http://raptorx.uchicago.edu).

### Quantification and statistical analysis

All statistical analyses were conducted by using GraphPad Prism software (GraphPad Software, San Diego, CA, USA), including unpaired, two-tailed Student’s *t*-tests, and one-way ANOVA at the 95% level with Tukey’s multiple comparison test. The values are presented as means ± standard deviation (SD).

## Acknowledgements

This work was supported by the National Natural Science Foundation of China (31872919).

## Author contributions

W.W. and H.Zo, X.C., and X.L. performed the research; T.Z. and X.F. helped with the data analysis; W.M. modified the manuscript. The final manuscript was approved by all the authors.

## Data availability

All relevant data are available within the article and its supplementary data.

## Conflict of interest statement

The authors declare that no competing interests exist.

## Supplementary data

Supplementary data is available at *Horticulture Research* online.

## Supplementary Material

Web_Material_uhae162
