# Supplementary material for: Ralstonia solanacearum type III effector RipAF1 mediates plant resistance signaling by ADP-ribosylation of host FBN1
Source: Hortic Res. 2024 Jun 12;11(8):uhae162. doi: 10.1093/hr/uhae162 (PMC11298624; doi:10.1093/hr/uhae162)
Supplement: Web_Material_uhae162 [file web_material_uhae162.zip › RipAF1_supple Fig-24.6.22.pdf]

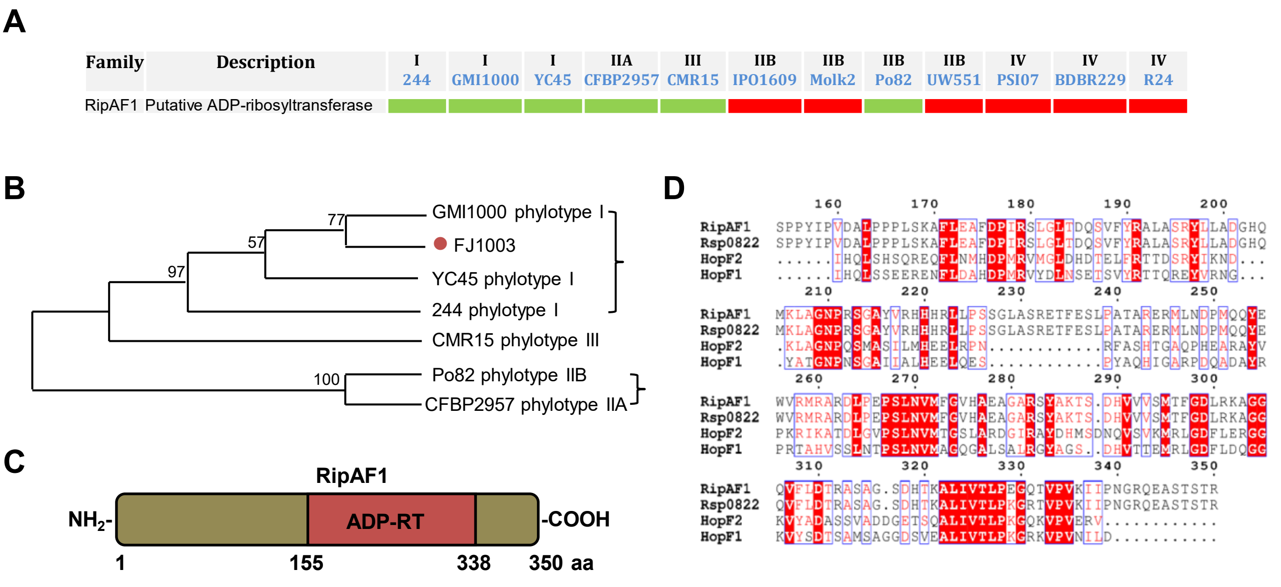

Figure S1 RipAF1 with an ADP-ribosyltransferase domain was conserved in *Ralstonia solanacearum*. **(A)** Distribution of RipAF1 in representative *R. solanacearum* species from four phylotypes. Green indicates one copy in the species, while red indicates the effector is missing. **(B)** Phylogenetic analysis of RipAF1 from FJ1003 and its homologs from other *R. solanacearum* species. The phylogeny was inferred using the maximum likelihood method implemented in MEGA 7.0. The bootstrap consensus tree inferred from 1000 replicates represents the evolutionary history of the taxa analyzed. **(C)** Schematic diagram of the RipAF1 structure possessing an ADP-ribosyltransferase (ADP-RT) domain. The ADP-RT domain from 155 to 338 residues is shown in red. **(D)** Amino acids sequence alignment of the ADP-RT domain in RipAF1 from FJ1003 and GMI1000, respectively, and HopF1 and HopF2 from *Pseudomonas syringae*. The sequences were aligned with Clustal X and analyzed using Espript3.0. The conserved amino acids are indicated in red.

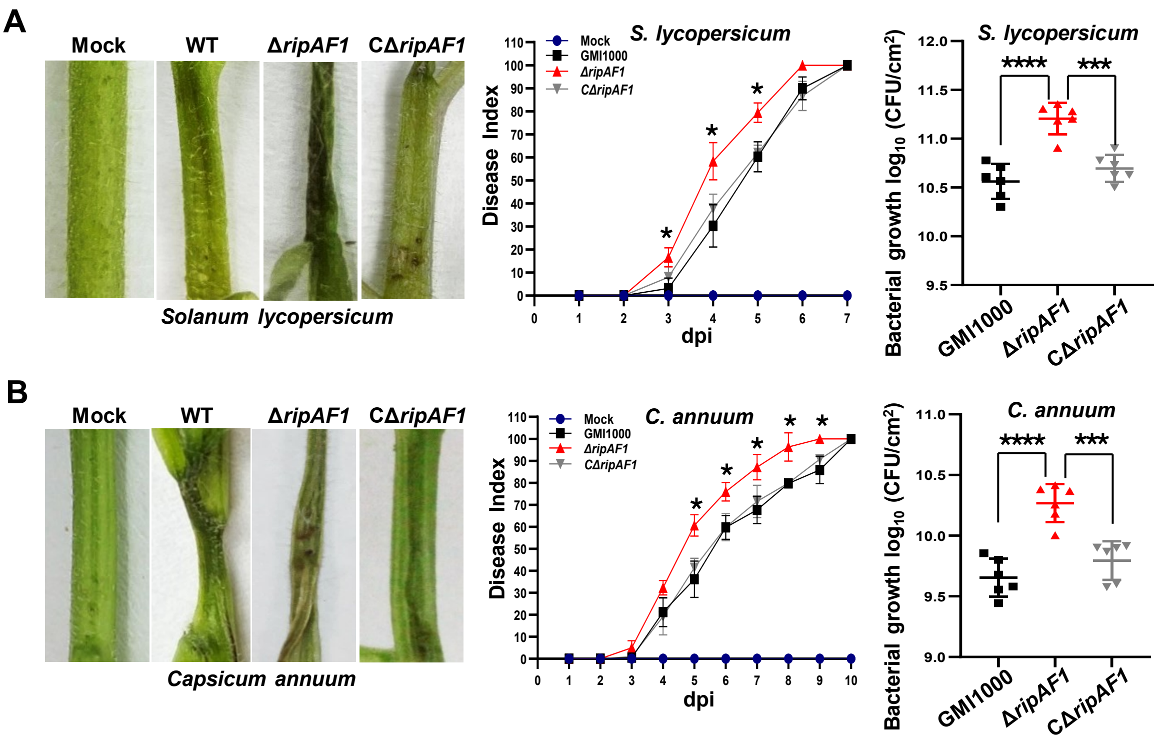

Figure S2 RipAF1 mutant of GMI1000 *Ralstonia solanacearum* showed enhanced virulence to *Solanum lycopersicum* and *Capsicum annuum*. **(A)** *S. lycopersicum* inoculated with wild-type GMI1000, Δ*ripAF1*, and CΔ*ripAF1*. Disease symptoms on stem tissue near inoculation sites were captured 7 d post-inoculation (dpi). All experiments were replicated three times with similar results, and representative results are shown. Disease severity was assessed after stem inoculation. Each time point represents the mean disease severity of 24 inoculated plants per treatment. Error bars represent the standard deviation of three independent experiments (two-way ANOVA, $*p < 0.05$, $**p < 0.01$). Bacterial growth was assessed on four-week-old *S. lycopersicum* plants at 3 days post inoculation with 100 μL of 10^6 CFU/mL bacterial cell suspension. Values are means ± SD ($n = 6$ biological replicates; Student's *t*-test, $***p < 0.001$, $****p < 0.0001$). **(B)** *C. annuum* plants inoculated with wild-type GMI1000, Δ*ripAF1*, and CΔ*ripAF1*, characterized as in (A).

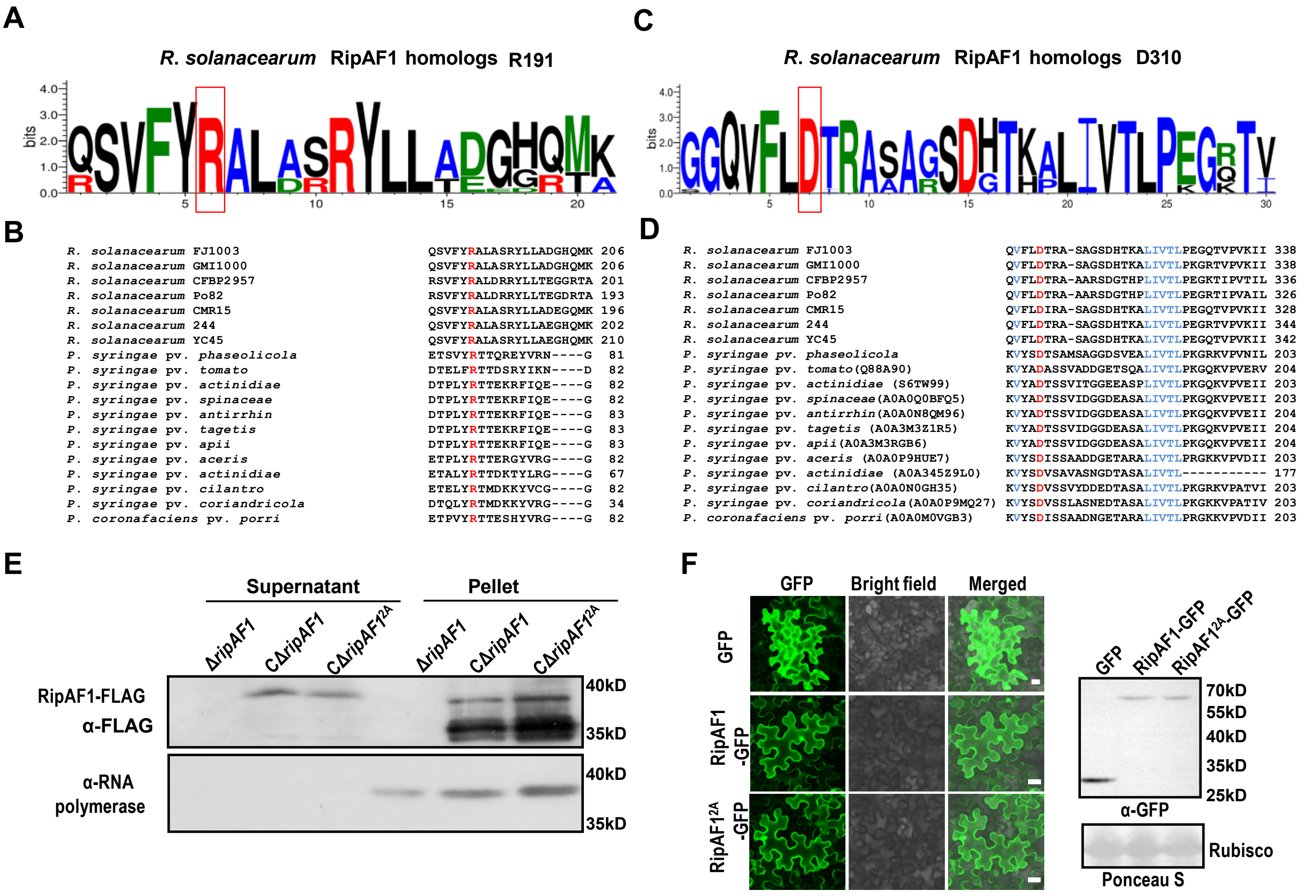

Figure S3 The Arg191/Asp310 residues among RipAF1 homologs. **(A)** WebLogo analysis of the conservation of the R191 residue in RipAF1 from *Ralstonia solanacearum.* Sequences were downloaded from Swiss-Prot and aligned with Clustal X. Aligned sequences were subjected to online WebLogo analysis. The conserved arginine acid (R191) is highlighted in red. **(B)** Multiple sequence alignment of the 22-amino acid domain including the R191 residue in RipAF1 homologs. Sequences of RipAF1 were downloaded from Swiss-Prot. Different pathovars of *Pseudomonas syringae* and other species from the genus *Pseudomonas* were selected for multiple sequence alignment by Clustal X. The conserved R191 is highlighted in red. **(C)** WebLogo analysis of the conservation of aspartic acid (D310) in RipAF1 from *R. solanacearum.* The analyses were performed as in (A). **(D)** Multiple sequence alignment of the 33-amino acid domain including the D310 residue in RipAF1 homologs. The conserved D310 is highlighted in red. Other invariant residues are shown in blue. The analyses were performed as in (B). **(E)** Secretion analysis of RipAF1^{2A} in Δ*ripAF1* complemented strains. The RipAF1 and RipAF1^{2A} fused with FLAG tag were expressed in Δ*ripAF1* and cultured in M63 medium. Bacterial polymerase protein levels served as the internal controls. Similar results were obtained from three independent experiments. **(F)** The subcellular localization of RipAF1^{2A}. RipAF1^{2A}-GFP was transiently expressed in *Nicotiana benthamiana* leaves following *Agrobacterium*-mediated transformation. The fluorescence was visualized by confocal microscopy. GFP and RipAF1-GFP were used as controls. Scale bar, 25 µm. The gels at right show the expression of GFP, RipAF1-GFP, and RipAF1^{2A}-GFP in western blots using anti-GFP antibody.

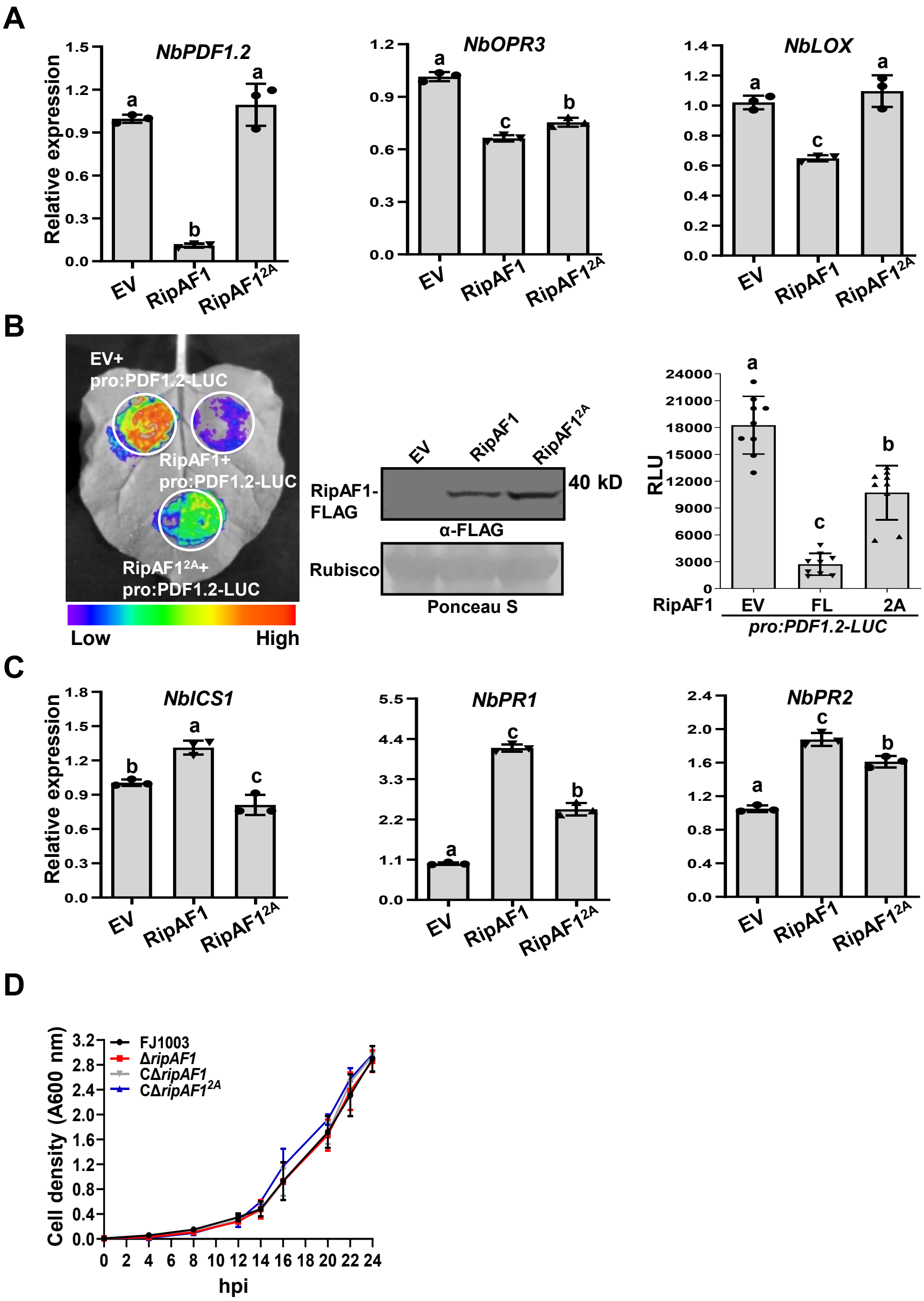

Figure S4 The Arg191/Asp310 residues in RipAF1 were critical for modulating jasmonic acid (JA) and salicylic acid (SA) signaling marker genes. **(A)** RipAF1^{2A} did not suppress the expression levels of JA marker genes *NbPDF1.2*, *NbOPR3*, and *NbLOX* in *N. benthamiana*. Total RNA was isolated from leaves 48 h after agroinfiltration. Expression levels were determined by qRT-PCR analysis and normalized to that of the empty vector pJL12 (EV). Columns labeled with the same letter indicate means were not significantly different. Values are means ± SD (n = 3 biological replicates; ANOVA with Tukey's test, $p < 0.01$). **(B)** RipAF1^{2A} exhibited a reduced ability to inhibit expression of *PDF1.2* promoter activity relative to wild-type RipAF1. The pro:PDF1.2-Luc construct was co-expressed with RipAF1-FLAG or RipAF1^{2A}-FLAG in *N. benthamiana* leaves. Luciferase activity was measured with a CCD imaging system. The co-expression of pro:PDF1.2-Luc with empty vector was used as the negative control. The gels show the expression of respective proteins. Quantitative assays of *PDF1.2* promoter activity when co-expressed with RipAF1 or RipAF1^{2A}. Quantification of the luciferase signal was performed with a microplate luminescence reader. Values are means ± SD (n = 9 biological replicates). Columns labeled with different letters represent significantly different means (ANOVA with Tukey's test, $p < 0.01$). **(C)** RipAF1^{2A} cannot increase the expression level of the SA signaling marker genes *NbICSI*, *N*b*PR1*, and *NbPR2* in *N. benthamiana*. performed as in (A). Values are means ± SD (n = 3 biological replicates; ANOVA, $p < 0.01$). All experiments were replicated three times with similar results, and representative results are shown. **(D)** Bacterial growth in nutrient-rich medium. FJ1003, Δ*ripAF1*, CΔ*ripAF1* and CΔ*ripAF1^{2A}* strains were inoculated into the complete NB liquid medium with initial OD_{600} = 0.01 and the bacterial growth was monitored at the indicated time points measuring OD_{600}, Values are means ± SD (n = 3 biological replicates).

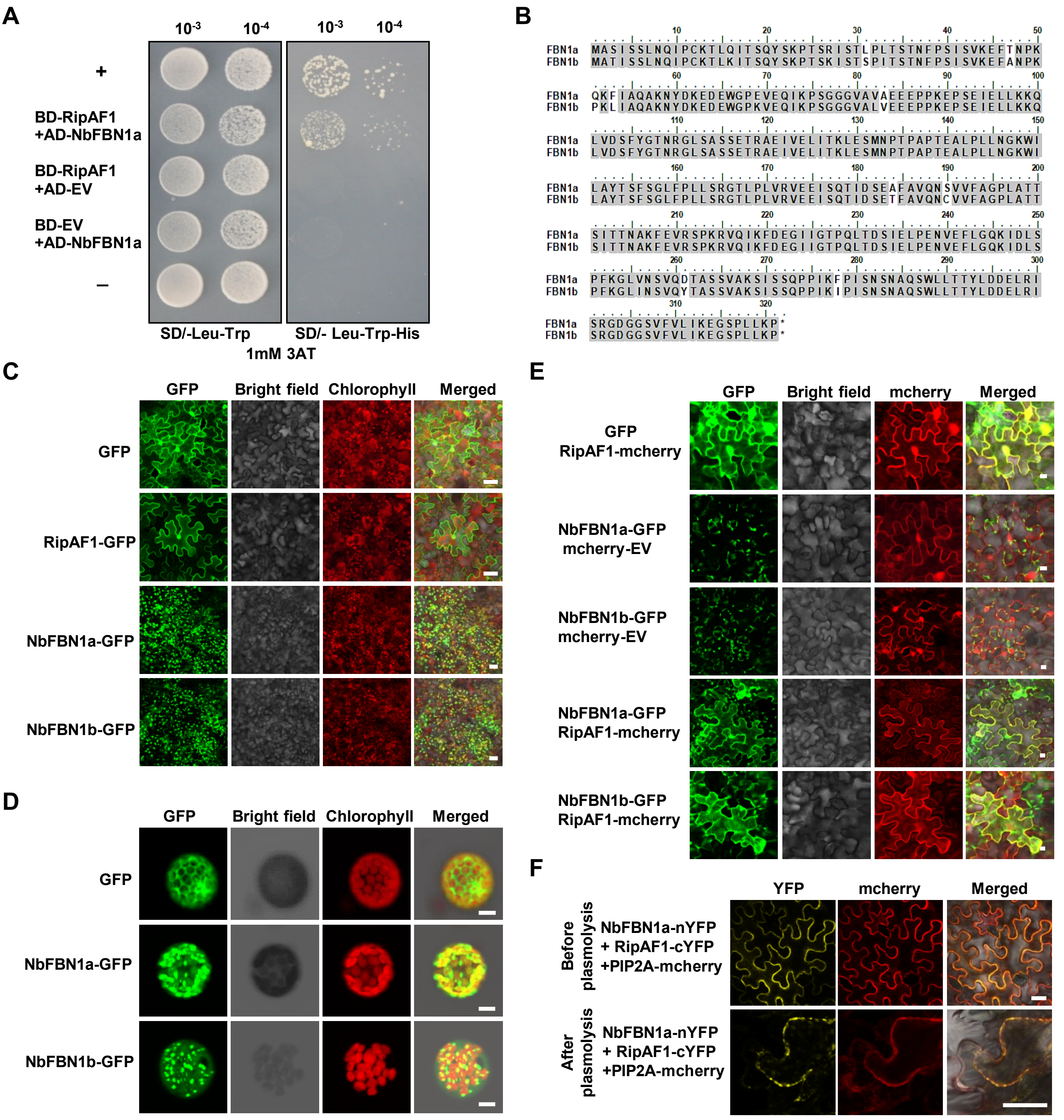

Figure S5 RipAF1 interacts with NbFBN1a/b at the cell membrane. **(A)** Growth of yeast co-transformed with BD-RipAF1 and AD-NbFBN1a on synthetic dextrose (SD) plates. The transformants were screened on SD media lacking leucine and tryptophan (SD/-Leu-Trp). The single yeast colonies were serially diluted and grown on SD/-Leu-Trp and SD/-Leu-Trp-His (SD media lacking leucine, tryptophan, and histidine) to examine the putative interaction. One mM 3-amino-1,2,4-triazole (3-AT) was supplied to inhibit the autoactivation of AD-NbFBN1a. Yeast co-transformed with pGADT7-T and pGBKT7-53 served as the positive control (+), and yeast co-transformed with pGADT7-T and pGBKT7-lam served as the negative control (-). EV, empty vector. **(B)** Amino acid sequence alignment of NbFBN1a and NbFBN1b. The sequences were aligned with Clustal X. **(C)** Subcellular localization of RipAF1-GFP and NbFBN1a/b-GFP. RipAF1-GFP and NbFBN1a/b-GFP fusions were transiently expressed in *N. benthamiana* leaves following *Agrobacterium*-mediated transformation. The images were captured using a confocal microscope. Scale bar, 25 μm. **(D)** NbFNB1a/b is a chloroplast-localized protein in the protoplast of *N. benthamiana*. Plasmids containing *35S:GFP* and *35S:NbFBN1a/b-GFP* were transformed into *N. benthamiana* protoplasts. Confocal micrographs were obtained 16 h post-transformation. Scale bar, 10 μm. **(E)** NbFNB1a/b and RipAF1 were co-localized to the cell membrane in *N. benthamiana*. NbFNB1a/b-GFP and RipAF1-mcherry were co-expressed in *N. benthamiana* and visualized by confocal microscopy. GFP co-expressed with RipAF1-mcherry and NbFBN1a/b-GFP co-expressed with mcherry-EV were used as the negative controls. Scale bar, 10 μm. **(F)** NbFBN1a/b interacted with RipAF1 in cell membranes of *N. benthamiana* leaves. NbFBN1a/b was fused with nYFP, and RipAF1 was fused with cYFP. The PIP2A-mcherry construct was used as a plasma membrane marker. The plasmolysis was performed by treatments with 10 mM NaCl for 5 min. Images were captured using a confocal microscope at 48 hpi. Scale bar, 25 μm.

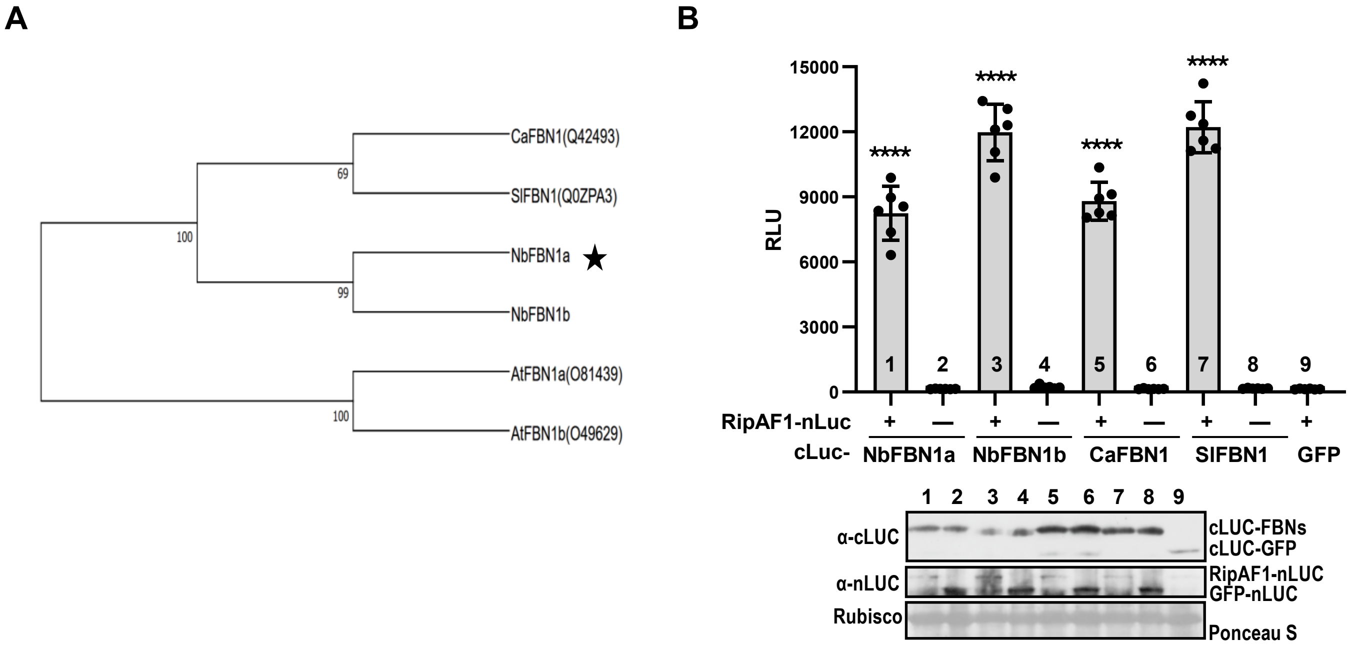

Figure S6 RipAF1 interacted with FBN1 from *Solanum lycopersicum* and *Capsicum annuum*. **(A)** The phylogeny based on FBN1 sequences from *Nicotiana benthamiana* (*Nb*), *Solanum lycopersicum* (*Sl*), *Capsicum annuum* (*Ca*), and *Arabidopsis thaliana* (*At*). The phylogenetic tree was generated using MEGA 7.0. The star indicates NbFBN1a from *N. benthamiana*. **(B)** Split luciferase assay to assess the interaction of RipAF1 with SlFBN1 and CaFBN1. GFP-nLUC served as the negative control (-). The protein interaction strength was quantified according to the luminescence signals. Values are means ± SD (n = 6 biological replicates; Student's t-test, $****p < 0.0001$). Western blotting was used to confirm the expression of the respective proteins.

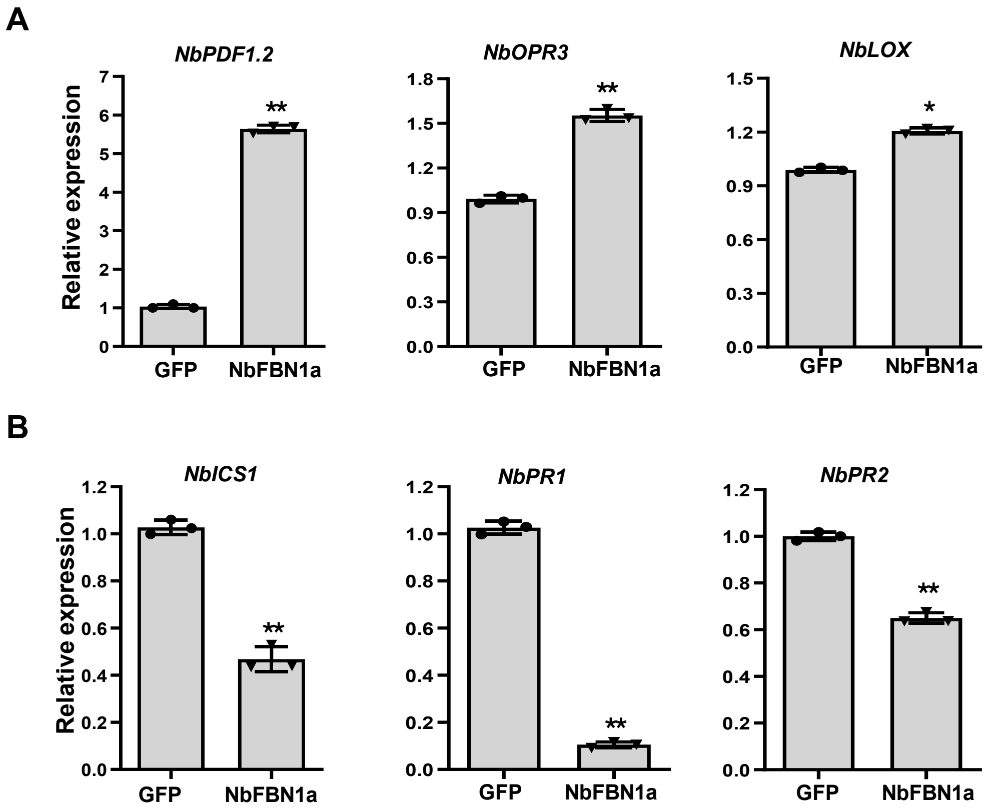

Figure S7 Transient overexpression of NbFBN1a activated JA and suppressed SA signaling in *Nicotiana benthamiana.* **(A)** NbFBN1a enhanced the expression of JA marker genes. Total RNA was isolated from leaves 48 h after agroinfiltration. Expression levels were determined by qRT-PCR analysis and normalized to that of the GFP control. Values are means ± SD (n = 3 biological replicates; Student's t-test, *$p < 0.05$, **$p < 0.01$. **(B)** NbFBN1a suppressed the expression of SA marker genes, based on the same procedure as in (A). Values are means ± SD (n = 3 biological replicates; Student's t-test, **$p < 0.01$).

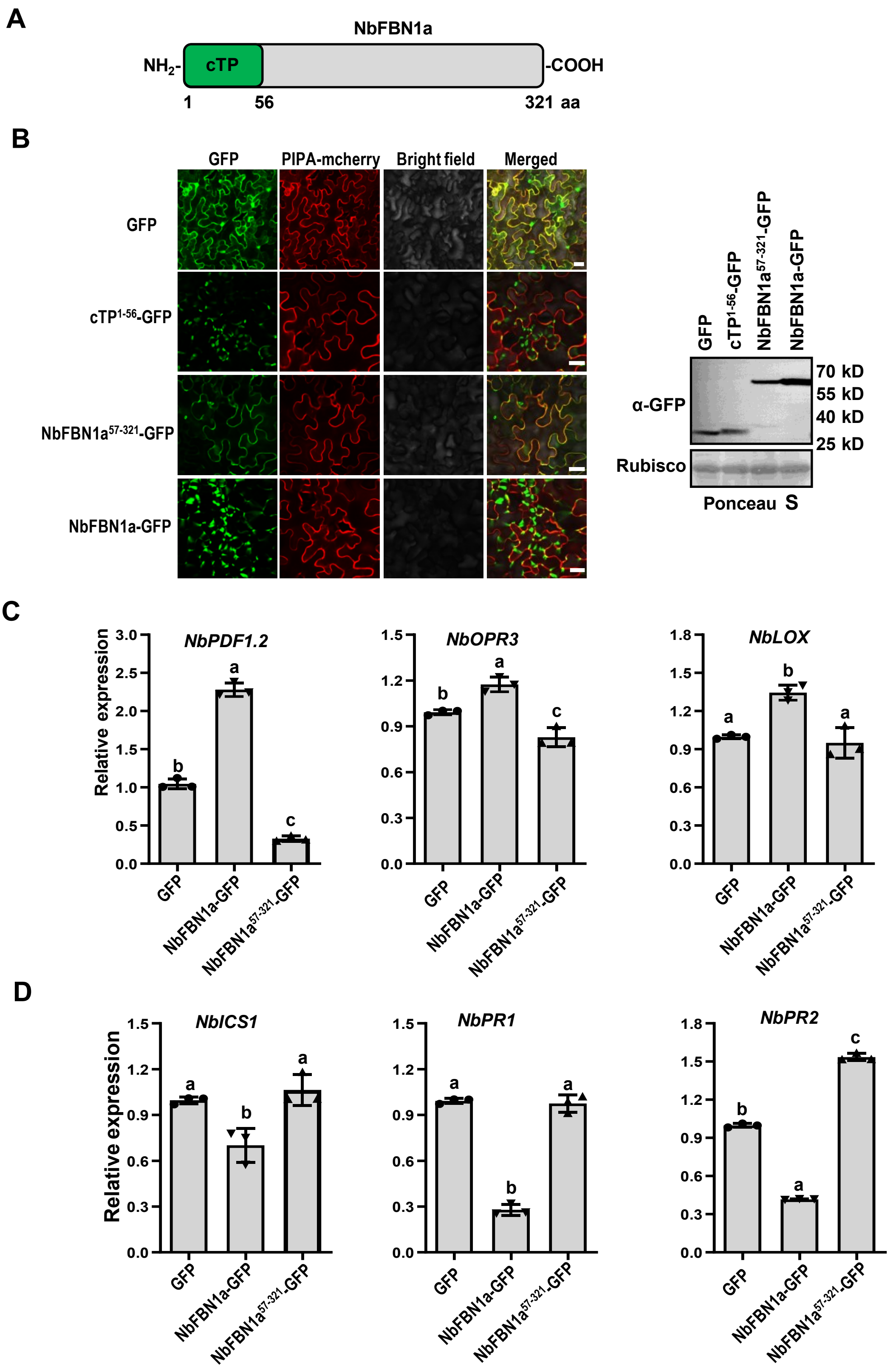

Figure S8 Location of NbFBN1a on chloroplast exerted a contrary role in JA and SA signaling from location at cell membrane. **(A)** Schematic diagram of the NbFBN1a structure with a chloroplast transit peptide (1-56 aa) by the LOCALIZER program. **(B)** Examination of subcellular location of cTP-GFP, NbFBN1a$^{57\text{-}321}$-GFP, and NbFBN1a-CFP in *Nicotiana benthamiana* cells when transiently co-expressed with a membrane location marker. Bar = 25 μm. The expression of corresponding proteins were detected with anti-GFP by western blot analysis. **(C)** The expression of JA signaling marker genes. Total RNA was isolated from leaves 48 h after agroinfiltration. Expression levels were determined by qRT-PCR analysis and normalized to that of the GFP control. Values are means ± SD (n = 3 biological replicates; Student's t-test, *$p < 0.05$, **$p < 0.01$. **(D)** The expression of SA signaling marker genes, based on the same procedure as in (A). Values are means ± SD (n = 3 biological replicates; Student's t-test, **$p < 0.01$).

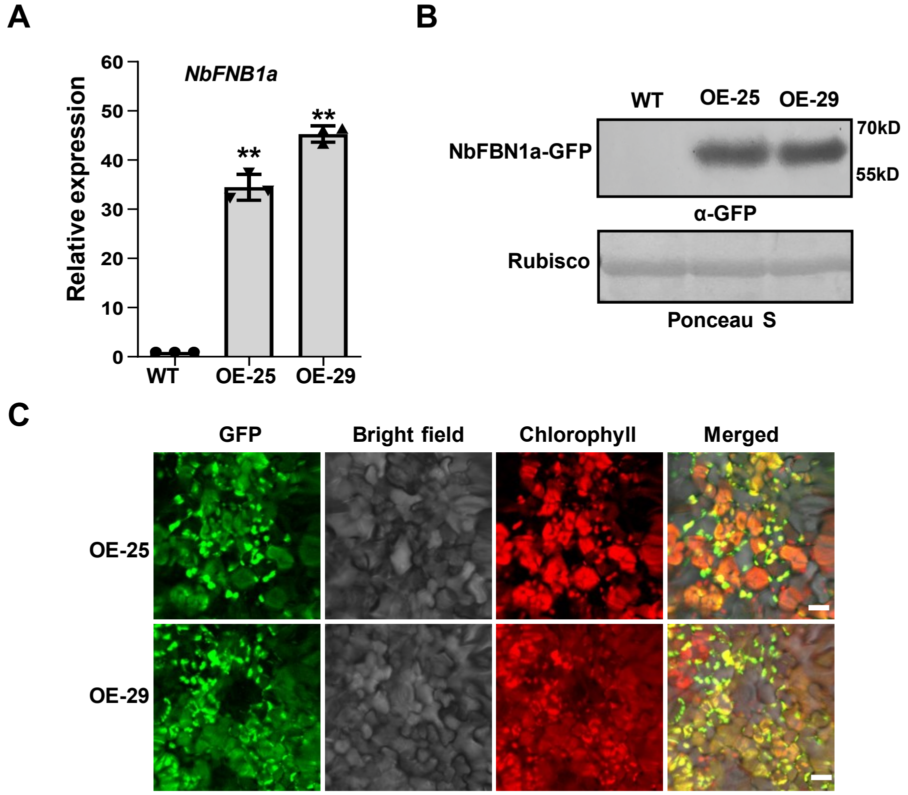

Figure S9 Detection of *NbFBN1a* expression in transgenic *Nicotiana benthamiana* plants. **(A)** Quantitative reverse transcription PCR analysis of the *NbFBN1a* transcript level in transgenic plants. Total RNA was isolated from four-week-old transgenic plants. The transcript level in wild-type *N. benthamiana* plants was used as a control to monitor expression changes. OE-25 and OE-29 are independent transgenic lines. Values are means ± SD (n = 3 biological replicates; Student's t-test, $^{**}p < 0.01$). **(B)** Western blot analysis of overexpression of NbFBN1a. The NbFBN1a-GFP transgenic lines OE-25 and OE-29 were assayed by anti-GFP immunoblotting. Wild-type *N. benthamiana* served as the negative control. **(C)** Examination of plastid-localized NbFBN1a-GFP in transgenic plant leaves. The images were captured using a confocal microscope. Scale bar, 25 μm.

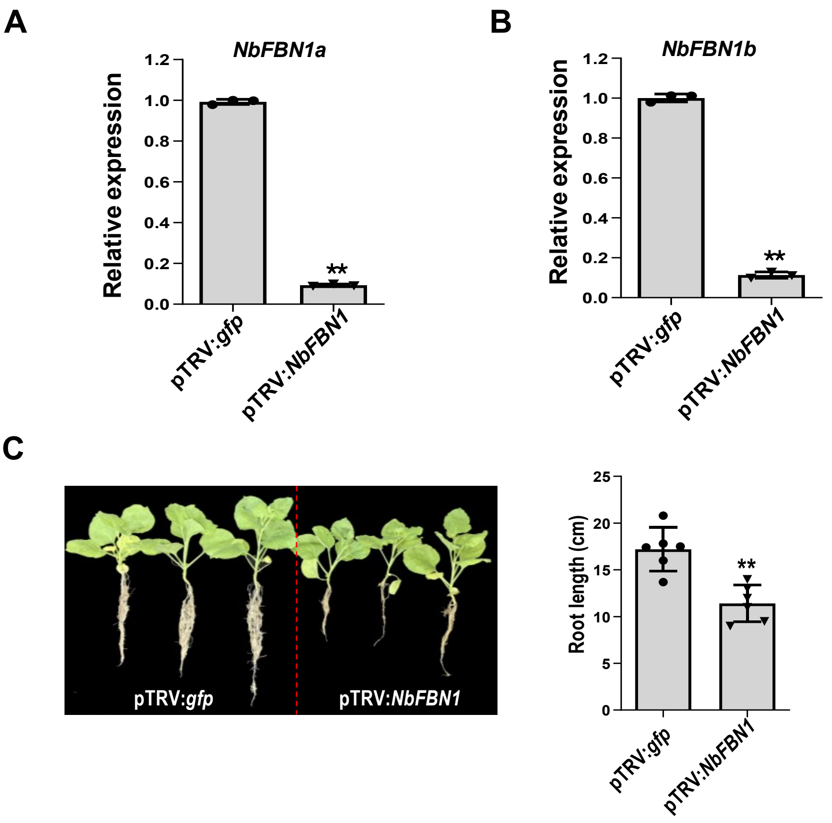

Figure S10 Assays of *NbFBN1*-silenced *Nicotiana benthamiana*. **(A)** Quantitative reverse transcription PCR analysis of the transcript level of *NbFBN1a* in *NbFBN1*-silenced plants. RNA was isolated from the new upper leaves when photobleaching was observed in phytoene desaturase (*PDS*)-silenced positive control plants. The transcript level in plants transformed with pTRV:*gfp* was used as a control to monitor expression changes. Error bars represent the standard deviation from three replicates. Values are means ± SD (n = 3 biological replicates; Student's t-test, $^{**}p < 0.01$). **(B)** Quantitative reverse transcription PCR analysis of the transcript level of *NbFBN1b* in *NbFBN1*-silenced plants. **(C)** The growth of plants transformed with pTRV:*NbFBN1*. The plants were collected at 3 weeks post-agroinfiltration

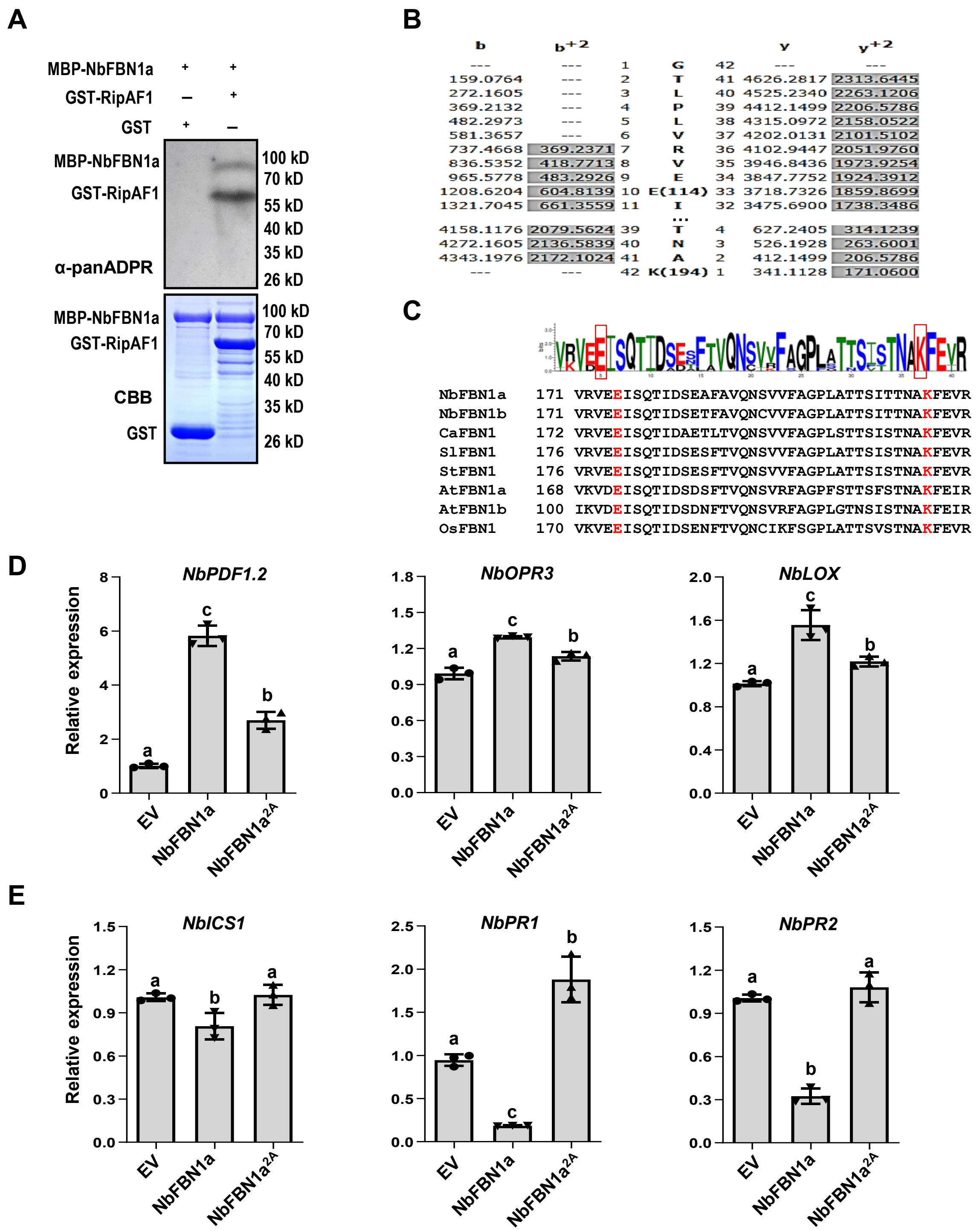

Figure S11 E175/K207 residues in NbFBN1a. **(A)** An *in vitro* ADP-ribosylation assay was conducted using recombinant proteins extracted from *E. coli*. These recombinant proteins were mixed in an ADPRT reaction buffer for the reaction. Biotin-NAD$^+$ was used as the ADP-ribose donor. The level of protein ADP-ribosylation was detected with anti-panADPR antibody. **(B)** The mass spectrometry of NbFBN1a modification peptide sequence was analyzed by MS-product (https://prospector.ucsf.edu/prospector/cgi-bin/msform.cgi?form=msproduct). The numbers 114 and 194 behind E and K represent the molecular weight of the modifications. **(C)** WebLogo analysis of the conservation of E175/K207 in FBN1 from *Solanum lycopersicum* (*Sl*), *Solanum tuberosum* (*St*), *Capsicum annuum* (*Ca*), *Arabidopsis thaliana* (*At*), and *Oryza sativa* (*Os*). FBN1 sequences were downloaded from Swiss-Prot and aligned with Clustal X. Aligned sequences were subjected to online WebLogo analysis. The conserved glutamic acid (E175) and lysine (K207) residues are highlighted in red. **(D)** NbFBN1a^{2A} attenuated activation of JA marker genes *NbPDF1.2*, *NbOPR3*, and *NbLOX*. Total RNA was isolated from four-week-old *Nicotiana benthamiana* plants 48 h after agroinfiltration. Expression levels were determined by qRT-PCR analysis and normalized to that of the empty vector plants. Columns labeled with the different letter indicate significant difference among means. Values are means ± SD (n = 3 biological replicates; ANOVA Tukey's test, $p < 0.01$). **(E)** NbFBN1a^{2A} did not influence SA marker gene *NbICS1*, *NbPR1*, and *NbPR2* in *N. benthamiana*, performed as in (D). Columns labeled with the same letter indicate means were not significantly different. Values are means ± SD (n = 3 biological replicates; ANOVA Tukey's test, $p < 0.01$).

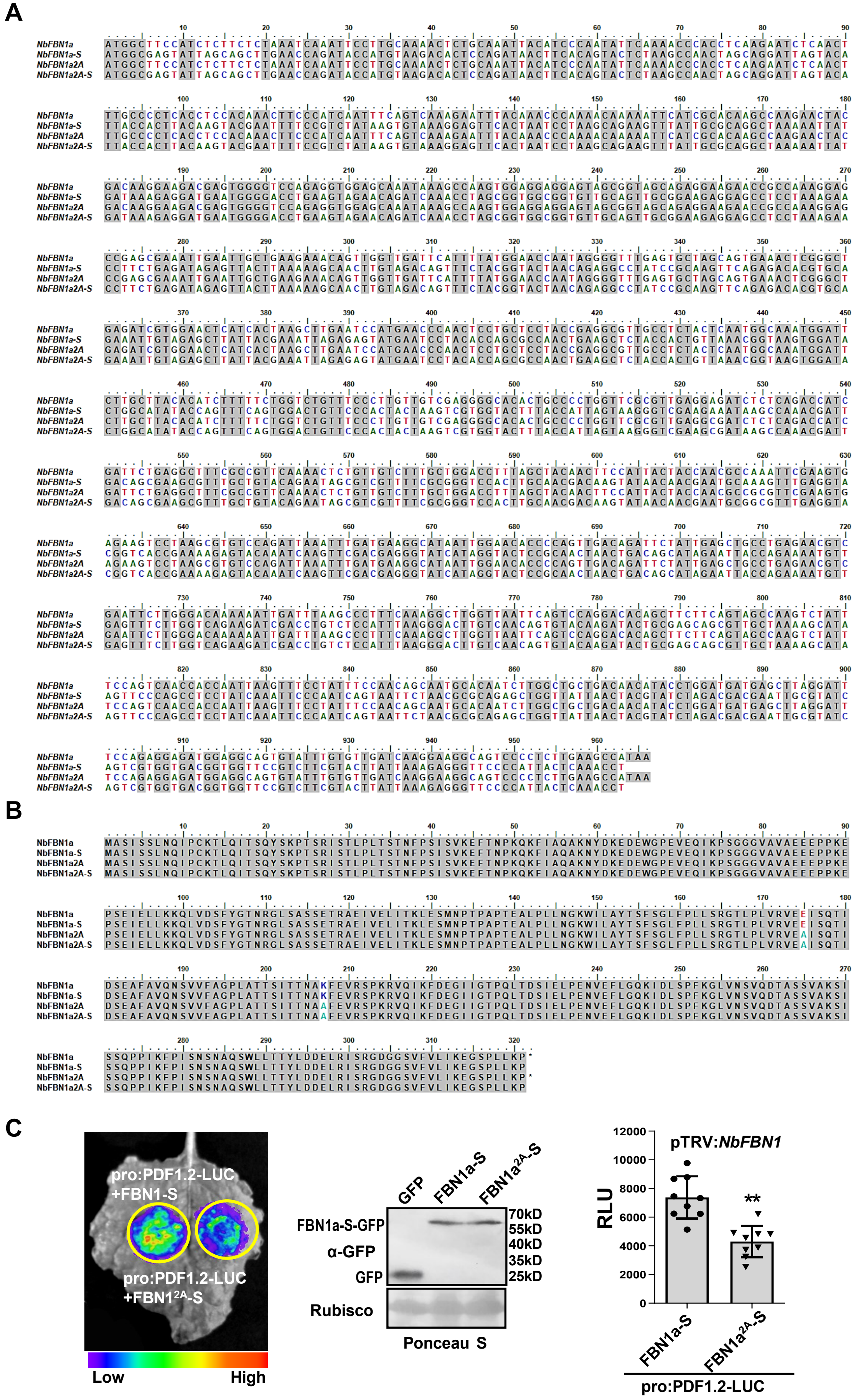

Figure S12 The effect of synthetic *NbFBN1a-S* and *NbFBN1a^{2A}-S* on *PDF1.2* promoter activity in *NbFBN1*-silenced plants. **(A)** Alignment of nucleotide sequences of *NbFBN1a*, *NbFBN1a^{2A}*, *NbFBN1a-S*, and *NbFBN1a^{2A}-S* showing the mutated sites . **(B)** Alignment of amino acid sequences of NbFBN1a, NbFBN1a^{2A}, NbFBN1a-S, and NbFBN1a^{2A}-S. **(C)** Luciferase assays of *PDF1.2* promoter activity affected by synthetic NbFBN1a-S and NbFBN1a^{2A}-S in pTRV:*NbFBN1* plants. Luciferase activity was measured with a CCD imaging system. Quantification of the luciferase signal was performed with a microplate luminescence reader. Values are means ± SD (n = 9 biological replicates; Student's t-test, **p < 0.01).

Table S1: Peptide information with ADP-ribosylation sites of NbFBN1a identified by mass spectrometry.

Peptide	Modifications	q-Value	Charge	MH+ [Da]	ΔM [ppm]
TLQITSQYSKPTSRISTLPLTST nFPPsISVKEFTNPk	1x$C_5H_7O_6P$ [S27]; 2x$C_5H_6O_3$ [N24; K37]	0	6	756.54315	-6.68
NYDKEDEWGPEVeQIKPSGG GVAVAeEEPPK	2xADP-Ribosyl [E13; E26]	0	1	4463.82259	-1.89101
LEsmNPtPAPTEALPLLNGKWI LAYTsFSGLFPLLSR	1xOxidation [M4]; 2x$C_5H_7O_6P$ [S3; S27]; 1x$C_5H_6O_3$ [T7]	0	6	761.37048	2.88
LESmNPTPAPTeALPLLNGk	1xOxidation [M4]; 1x$C_5H_7O_6P$ [K20]; 1x$C_5H_6O_3$ [E12]	0	2	1209.07446	9.22
GTLPLVRVEeISQTIDSEAFAV QNSVVFAGPLATTSITTNAk	1x$C_5H_7O_6P$ [K42]; 1x$C_5H_6O_3$ [E10]	0	6	781.39801	0.00811
VQIKFdEGIIGTPQLTDSIeLPE NVEFLGQKIDLSPFK	1x$C_5H_7O_6P$ [D6]; 1x$C_5H_6O_3$ [E20]	0	6	761.87695	-12.64
VQIKFdEGIIGtPQLtDSIELPEN VEFLGQK	1xADP-Ribosyl [T12]; 1x$C_{10}H_{16}O_{13}P_2$ [T16]; 1x$C_5H_7O_6P$ [D6]	0	5	920.58368	2.92
FPISnSNAQSWLLTTYLDDeLR ISRGDGGSVFVLIk	1x$C_5H_7O_6P$ [S/N/K/D/R/T]; 3x$C_5H_6O_3$ [N5; E20; K36]	0	6	758.8761	10.04

Note:Recombinant protein His-MBP-NbFBN1a and GST-RipAF1 were co-expressed in *E. coil*. Trypsin-digested recombinant proteins were subjected to LC-MS/MS analysis. Mass spectrometry raw data was subjected to search database using Mascot software. His-MBP-NbFBN1a co-expression with GST-RipAF1^{2A} was served as negative control. Modification sites were highlighted in red. Modification sites all were not identified in the negative control. The ppm represents part per million.

Table S2. Primers used in this study.

Gene ID	primer (5'→3')
**Primers used for gene cloning**	
RipAF1-CDS-F	ATGGGTTTGCCACGGATCC
RipAF1-CDS-R	TCATCGCGTTGACGTGGAC
NbFBN1a-CDS-F	ATGGCTTCCATCTCTTCTCT
NbFBN1a-CDS-R	AGGCTTCAAGAGGGGACTGC
NbFBN1b-CDS-F	ATGGCTACCATCTCTTCTCT
NbFBN1b-CDS-R	AGGCTTCAAGAGGGGACTGC
**Primers used for point mutation**	
RipAF1-R191A-F	GGGCCTGACGGACCAGTCCG TCTTCTAC **GCG** GCGCTCGCT
RipAF1-R191A-R	AGCGAGCGC **CGC** GTAGAAGACGG
RipAF1-D310A-F	CAAGGCAGGCGGACAG GTCTTTCTC **GCG** ACGCGCGCCTC
RipAF1-D310A-R	GAGGCGCGCGT **CGC** GAGAAAGAC
NbFBN1a-E175A-F	GGGCACACTGCCCCTGGTT CGCGTTGAG **GCG** ATCTCTCAGAC
NbFBN1a-E175A-R	GTCTGAGAGAT **CGC** CTCAACGCG
NbFBN1a-K207A-F	GCTACAACTTCCATTA CTACCAACGCC **GCG** TTCGAAGTGAG
NbFBN1a-K207A-R	CTCACTTCGAA **CGC** GGCGTTGGTAG
**Primers used for protein expression**	
GST-RipAF1-F	ATCTGGTTCCGCGTGGATCC ATGGGTTTGCCACGGATCCC
GST-RipAF1-R	TCACGATGCGGCCGCTCGAG TCATCGCGTTGACGTGGACG
His-MBP-RipAF1-F	GGATTTCAGAATTCGGATCC ATGGGTTTGCCACGGATCCC
His-MBP-RipAF1-F	TGGTGGTGGTGGTGCTCGAG TTATCGCGTTGACGTGGACGCCT
GST-NbFBN1a-F	ATCTGGTTCCGCGTGGATCC ATGGCTTCCATCTCTTCTCT
GST-NbFBN1a-R	TCACGATGCGGCCGCTCGAG TTA TGGCTTCAAGAGGGGACTGC
His-MBP-NbFBN1a-F	GGATTTCAGAATTCGGATCC ATGGCTTCCATCTCTTCTCT
His-MBP-NbFBN1a-F	TGGTGGTGGTGGTGCTCGAG TTATGGCTTCAAGAGGGGAC
MBP-NbFBN1a-F	GGATTTCAGAATTCGGATCC ATGGCTTCCATCTCTTCTCT
MBP-NbFBN1a-F	AAGCTTGCCTGCAGGTCGAC AGGCTTCAAGAGGGGACTGC
**Primers used for BiFC**	
nYFP-NbFBN1a-F	GCTTCGAATTCTGCAGTCGAC ATGGCTTCCATCTCTTCTCT
nYFP-NbFBN1a-R	ACTCTAGATCAGGTGGATCC CTA TGGCTTCAAGAGGGGACTGC
cYFP-RipAF1-F	CTTCGAATTCTGCAGTCGAC ATGGGTTTGCCACGGATCCC
cYFP-RipAF1-R	ACTCTAGATCAGGTGGATCC CTA TCGCGTTGACGTGGACGCCT
**Primers used for split luciferase**	
Nluc-RipAF1-F	GAGCTCGGTACCCGGGATCC ATGGGTTTGCCACGGATCC
Nluc-RipAF1-R	GCGTACGAGATCTGGTCGAC TCGCGTTGACGTGGACGCCT
Cluc-NbFBN1a-F	CGGGGCGGTACCCGGGATCC AATG ATGGCTTCCATCTCTTCTCT
Cluc-NbFBN1a-R	CGAAAGCTCTGCAGGTCGAC TTA AGGCTTCAAGAGGGGACTGC
Cluc-NbFBN1b-F	CGGGGCGGTACCCGGGATCC AATG ATGGCTACCATCTCTTCTCT
Cluc-NbFBN1b-R	CGAAAGCTCTGCAGGTCGAC TTA AGGCTTCAAGAGGGGACTGC
**Primers used for yeast two hybrid**	
BD-RipAF1-F	TCAGAGGAGGACCTGCAT ATGGGTTTGCCACGGATCCC
BD-RipAF1-R	CGACGGATCCCCGGGAATTC TCATCGCGTTGACGTGGACG
AD-RipAF1-F	GTACCAGATTACGCTCAT ATGGGTTTGCCACGGATCCC
AD-RipAF1-R	TGCCCACCCGGGTGGAATTC TCATCGCGTTGACGTGGACG

**Primers used for yeast two hybrid**		
BD-NbFBN1a-F	TCAGAGGAGGACCTGCAT	ATGGCTTCCATCTCTTCTCT
BD-NbFBN1a-R	CGACGGATCCCCGGGAATTC	TTAAGGCTTCAAGAGGGGAC
AD-NbFBN1b-F	GTACCAGATTACGCTCAT	ATGGCTTCCATCTCTTCTCT
AD-NbFBN1b-R	TGCCCACCCGGGTGGAATTC	TTAAGGCTTCAAGAGGGGAC
**Primers used for localization**		
RipAF1-GFP-F	AAGATCTCGAGCTCAAGCTT	ATGGGTTTGCCACGGATCC
RipAF1-GFP-R	ACTCATGGGCCCGTCGAC	TCGCGTTGACGTGGACGCCT
GFP-RipAF1-F	AGATCTCGAGCTCAAGCTTCG	ATGGGTTTGCCACGGATCC
GFP-RipAF1-R	CCGCCCGCGGTACCGTCGAC	TCGCGTTGACGTGGACGCCT
RipAF1-mcherry-F	TCGAGCTCAAGCTTGTCGAC	ATGGGTTTGCCACGGATCC
RipAF1-mcherry-R	ACCATTCCTCCGCCGGGCCC	TCGCGTTGACGTGGACGCCT
NbFBN1a-GFP-F	GCTTTCGCGAGCTCGGTACC	ATGGCTTCCATCTCTTCTCT
NbFBN1a-GFP-R	CCCTTGCTCACCATGGATCC	TGGCTTCAAGAGGGGACTGC
**Primers used for complementation**		
pBB5-RipAF1-F	TCGACGGTATCGAT AAGCTT	GAATCCCCGATGCTACGCA
pBB5-RipAF1-R	CCCCCGGGCTGCAG GAATTC	TCATCGCGTTGACGTGGAC
PBB5-RipAF1-FLAG-EcoRⅠ-R	CCCCCGGGCTGCAG GAATTC	TTACTTATCATCATCATCC
**Primers used for transient expression**		
RipAF1-FLAG-F	TCTGATCAAGAGACAGGATCC	ATGGGTTTGCCACGGATCC
RipAF1-FLAG-R	GTAGTCAGCGGCCGCTCTAGA	TCGCGTTGACGTGGACGCCT
**Primers used for VIGS**		
pTRV2-NbFBN1a-F	TGAGTAAGGTTACC GAATTC	ATTCATCGCACAAGCCAAGA
pTRV2-NbFBN1a-R	AGACGCGTGAGCTC GGTACC	GCAAGAATCCATTTGCCATT
**Primers used for qRT-PCR**		
RipAF1-qRT-F	GACAGGTCTTTCTCGACA	
RipAF1-qRT-R	CGTTCGGAATGATCTTGA	
NbFBN1a-qRT-F	TACAAACCCAAAACAAAAATTC	
NbFBN1a-qRT-R	CGGTTCTTCCTCTGCTAC	
NbFBN1b-qRT-F	CGCAAACCCAAAACCAAAATTG	
NbFBN1b-qRT-R	TGGTTCTTCCTCTACTAA	
NbPR1-qRT-F	ATGGTCAATACGGCGAAAAC	
NbPR1-qRT-R	CCTAGCACATCCAACACGAA	
NbPR2-qRT-F	CAGCAGCAGGGTTGCAAGAT	
NbPR2-qRT-R	TTTGGGCGGGTAGGTATTC	
NbICSI-qRT-F	GAGGAATGTATGCTGGTC	
NbICSI-qRT-R	CTTTTCAACCAAAGCTGA	
NbPDF1.2-qRT-F	CATGGCTACTACAGAGATG	
NbPDF1.2-qRTt-R	CACTCTAACACATGGTCC	
NbOPR3-qRT-F	CTTCATGTGACTCAGCCACGAT	
NbOPR3-qRT-F	ACGCGCTTCCTCCTCTTCAC	
NbLOX-qRT-F	GTCCTACATTAAGCCGAA	
NbLOX-qRT-R	TGTCCATTCAGGTGATTC	
